# The Role of Parental Education, Intelligence, and Personality on the Cognitive Abilities of Gifted Children

**DOI:** 10.3390/jintelligence13020012

**Published:** 2025-01-21

**Authors:** Lina Pezzuti, Morena Farese, James Dawe, Marco Lauriola

**Affiliations:** 1 Department of Dynamic and Clinical Psychology and Health Studies, Sapienza University of Rome, 00185 Rome, Italy; lina.pezzuti@uniroma1.it (L.P.); morena.farese@uniroma1.it (M.F.); 2 Department of Social and Developmental Psychology, Sapienza University of Rome, 00185 Rome, Italy; james.dawe@uniroma1.it

**Keywords:** gifted children, parental education, parental intelligence, parental personality traits, cognitive abilities, CHC theory, Five-Factor Model, working memory

## Abstract

Several studies have indicated that parental education predicts children’s intelligence. In contrast, fewer studies have simultaneously analyzed the role of parental intelligence, education, and personality in shaping their children’s giftedness. This study investigated the effects of parental education, cognitive abilities (based on CHC theory), and personality traits (based on the Five-Factor Model) on the expression of gifted children’s cognitive abilities. Sixty-five gifted children (IQ ≥ 120) aged 6 to 14 years (M = 9.91 years; SD = 2.24 years) were assessed using the WISC-IV, while parents (65 mothers, M = 44.00 years; SD = 4.20 years, and 61 fathers, M = 45.70 years; SD = 5.40 years) completed the WAIS-IV and the Big-Five Inventory. The results indicated that maternal education was a key predictor of children’s Verbal Comprehension Index (VCI) in bivariate analyses, though its effect was not robust in multivariate models. Children’s Perceptual Reasoning Index (PRI) was associated with maternal conscientiousness, and fathers’ short-term memory (Gsm) emerged as the primary predictor of children’s Working Memory Index (WMI). Maternal processing speed (Gs) was the strongest predictor of children’s Processing Speed Index (PSI) across both bivariate and multivariate analyses. While personality traits, such as maternal conscientiousness, played a role in facilitating visual-spatial reasoning, their effects were weaker compared to cognitive and educational factors. The findings obtained, which are only partly consistent with data in the literature, highlight the domain-specific influence of parental characteristics on children’s giftedness and underscore the need for further research into the interplay of genetic, cognitive, and environmental factors.

## 1. Introduction

There is a consensus that giftedness is the result of a complex interplay of genetic, personal, and behavioral characteristics that culminate in exceptional abilities, which can manifest in various ways across one or more prominent areas defined by one’s cultural context (e.g., Keating 2009; Pfeiffer 2012; Worrell and Erwin 2011). In the Western world, giftedness is expressed in five major areas: general intellectual ability, specific scholastic aptitudes, creative or productive thinking, leadership skills, and visual and performing arts or psychomotor abilities (Worrell and Erwin 2011).

### 1.1. The Evaluation of Giftedness in Children

Although IQ represents only a partial expression of giftedness, from a strictly psychometric perspective, giftedness is defined by an IQ of 130 or higher, placing individuals at least two standard deviations above the population mean (Morrone et al. 2019; Winner 2000). However, many authors have extended this range by stating that individuals with an IQ below 130 can also be considered talented and, “…mirroring degrees of intellectual disability, there are degrees of giftedness: mild, moderate, high, exceptional and profound. The mildly gifted range begins at 1 1/3 SD (120 IQ), at the 91st percentile” (Silverman 2018, p. 185). Not too dissimilar, Ruf (2009) also classifies gifted children as having IQ scores of at least 120 and calls them “moderately gifted”. The National Association for Gifted Children (NAGC 2025) defines giftedness as the top 10% of the population. Roughly equivalent to the “superior” range, children with approximately 119 IQ or higher comprise the pool addressed in most talent search models. Following this same line of thought, in Italy, individuals with IQ scores between 120 and 129 are referred to as having “high potential” (Sartori 2019; Zanetti 2017), as they demonstrate the potential to excel despite scoring below 130 on standardized tests.

Scholars have argued that full-scale IQ is not the best metric for capturing the complexity of gifted individuals’ intellectual abilities (Morrone et al. 2019; Rimm et al. 2008; Rowe et al. 2014; Silverman 2009; Sparrow et al. 2005). Some authors have suggested using the WISC-IV General Ability Index (GAI) to minimize the influence of the speed and memory subtests, where gifted children often perform below their average (Hagmann-von Arx et al. 2008; Rimm et al. 2008; Silverman 2009; Sparrow et al. 2005). For instance, an Italian study (Toffalini et al. 2017) involving a large sample of gifted children with specific learning disorders demonstrated that giftedness was more evident when the General Ability Index (GAI) was utilized. However, this perspective is not universally accepted. For example, Rowe et al. (2014) contended that, rather than aggregating scores, a more accurate representation of performance for many gifted individuals may be achieved by examining the four index scores independently.

While the debate continues regarding the most effective method for identifying giftedness, gifted children exhibit a range of characteristics beyond general intellectual ability alone. For example, it is widely agreed that gifted children exhibit broad interests and strong curiosity, empathy, leadership tendencies, engagement in challenging and/or self-interested situations, a strong sense of justice, and high energy (e.g., Clark 2001; Song and Porath 2006) and tend to demonstrate high task commitment and creativity (e.g., Renzulli’s (2011) three-ring model). However, as Silverman (2018, p. 188) recently stated, “No alternative method [to intelligence tests] has demonstrated greater predictive validity in identifying the gifted,” and Sparrow et al. (2005, p. 295) also affirmed, “The use of intelligence tests is a core tool, and justifiably so. In terms of reliability and validity, standardized intelligence tests are usually the most psychometrically sound instruments available”. These positions have also been reflected in the CNOP (2018, p. 6) guidelines for the Italian assessment of gifted children, which state that “these characteristics cannot be understood as identifying criteria, as there is no finding in the literature that supports the identification of giftedness based solely on the presence or absence of these characteristics”.

### 1.2. Children’s Giftedness and Parents’ Cognitive Abilities

Despite significant expansion in research on intellectual endowment in recent decades, there is still limited knowledge about the cognitive characteristics of the parents of gifted children. Galton’s early observations were followed by the development and implementation of increasingly well-designed studies with larger, more representative samples over the past century (Plomin and von Stumm 2018). These studies have led researchers to conclude that approximately half of the variability in human intelligence can be attributed to hereditary factors passed from one generation to the next (Deary et al. 2006, 2009; Guez et al. 2021; Plomin and von Stumm 2018). However, these studies have also demonstrated that, while hereditary factors account for most individual differences in cognitive development from adolescence into adulthood, environmental factors exert the greatest influence during the transition from childhood to adolescence (Bouchard 2013; Davis et al. 2009; Deary et al. 2009; Johnson 2010; Nisbett et al. 2012). Direct estimates of shared environmental influence are derived from correlations of 0.19 between adoptive parents and their adopted children and 0.32 between adoptive siblings (Deary et al. 2006; Plomin and Spinath 2004). Since adoptive siblings are genetically unrelated, their similarity is clearly the result of shared upbringing. This suggests that about one-third of the total variance in intelligence can be explained by shared environmental factors. However, despite many efforts to identify the specific environmental characteristics that impact cognitive development, these efforts have largely been unsuccessful. Nevertheless, the influence of certain socioeconomic variables cannot be entirely dismissed (Plomin and Spinath 2004).

Previous research showed that there is greater consistency in mother–child dyads compared to father–child dyads (Anger and Heineck 2010; Calderon and Hoddinott 2010; Demange et al. 2022; Grönqvist et al. 2010). Anger and Heineck (2010) found that the correlation of IQ between parents and children was approximately r ≈ 0.34 for father–daughter dyads and r ≈ 0.48 for mother–daughter dyads. Similarly, Grönqvist and colleagues (2010) reported father–son correlations of r ≈ 0.51, while mother–son correlations were approximately r ≈ 0.59. Grönqvist et al. (2010) also assessed parent–child similarities in personality traits and found that the correlations for mother–child and father–child dyads were more similar, at around r ≈ 0.46.

Beyond IQ, a few studies have identified significant associations within specific cognitive domains, such as maternal and child processing speed, as well as paternal and child working memory (Anger and Heineck 2010; Min et al. 2007).

### 1.3. Children’s Giftedness and Parents’ Education

Parents’ education is widely recognized as the single most important factor influencing children’s intellectual achievement and cognitive development (Brooks 2011; Cave et al. 2022; Cianci et al. 2013). This conclusion has been supported by numerous studies (Craig 2006; Duncan and Magnuson 2012; Meekes et al. 2015; Mercy and Steelman 1982; Rindermann and Baumeister 2015). A recent large-scale analysis of data from seven countries further reinforced this claim, demonstrating that parents’ education was more strongly associated with differences in children’s IQ than other socioeconomic factors, including household wealth (Rindermann and Ceci 2018). Children of highly educated parents consistently achieved higher IQ scores, a result attributed to a combination of genetic predispositions for learning and the supportive, stimulating learning environments fostered by their parents (Evans et al. 2010).

Parents’ education was found to directly predict the extent to which they provided opportunities for cognitive engagement with their children. Parents from highly educated backgrounds strived to create more cognitively stimulating environments (Biedinger 2011) and promote learning through high-quality and high-quantity child-directed language (Rowe 2012). College-educated mothers used more words, produced longer utterances, and provided more topic-continuing responses when talking to their children compared to high school-educated mothers (Hoff 2003). Furthermore, educated parents provide more books, learning materials, and educational activities for their children (Davis-Kean 2005) and spend more time engaging with them, offering greater levels of cognitive support and stimulation (Guryan et al. 2008).

The scientific literature makes it clear that both parents’ education is important for children’s outcomes. Traditionally, more attention has been given to the role of mothers’ education in shaping children’s IQ (Labin et al. 2015). Mothers are the primary caregivers and spend more time with their children than fathers (Carneiro et al. 2013; Harding 2015; Mercy and Steelman 1982). They exert a significant influence on the rearing environment. Mothers’ education is significantly associated with early childhood IQ even after controlling for other socioeconomic factors, such as household income (Brooks-Gunn and Duncan 1997).

In families where mothers work more hours and spend less time with their children, fathers could also take on the role of caregiver. This could make fathers’ education a relevant factor in shaping the child’s rearing environment (Ermisch and Pronzato 2010). Fathers’ education is also important in traditional families because it correlates with household income. Educated fathers typically secure higher-paying jobs, which allows families to better support their children’s cognitive development (Bradley and Corwyn 2002; Carlson and Corcoran 2001; Khanam and Nghiem 2016).

### 1.4. Children’s Giftedness and Parents’ Personality

There is a gap in research on the relationship between children’s giftedness and parents’ personality. Most studies are outdated, dating back to the 1980s and 1990s. In one of the earliest studies (Fell et al. 1984), 62 parents of gifted children completed Cattell’s 16 PF Questionnaire. The results showed that mothers of gifted children described themselves as more intelligent, conscientious, and persistent than the general female population. They also reported being more independent and self-sufficient, preferring to make their own decisions, and more self-controlled. Similarly, fathers of gifted children reported being more intelligent, independent, and self-sufficient than the general male population. They were also characterized as more aloof, reserved, and critical; more assertive, confident, and dominant; and more tense.

From a behavioral point of view, Loeb and Jay (1987) showed that mothers of gifted children valued independence, while mothers of non-gifted children prioritized obedience. Similarly, Karnes and Shwedel (1987) found that fathers of gifted children differed notably from their non-gifted counterparts. These fathers emphasized reading, shared diverse materials, and used oral language as a teaching tool. They also reinforced self-esteem by fostering a positive self-image and encouraging independence, trust, and curiosity in their children.

Two classical reviews (Boyle 1988; Gockenbach 1989) summarized the scattered findings regarding the personality characteristics of parents of gifted children. The data from these reviews are limited, but parents of gifted children tended to be more intelligent, critical, assertive, and have a strong commitment to supporting their own ideas. They also exhibited greater independence, self-reliance, conscientiousness, and perseverance, as well as higher levels of self-control.

A later study by Landau and Weissler (1993) found that parents of gifted children engaged in cognitive interactions marked by openness to diverse topics and the use of rich, complex language. These parents believed in exposing their children to new experiences, even if such experiences were challenging or unpleasant, and responded positively to their children’s efforts toward personal growth. This approach reflects traits associated with intellectual curiosity and a preference for novelty, commonly linked to the personality factor of Five-Factor Model Openness (see McCrae and Costa 2010). Consistent with earlier findings (Boyle 1988; Fell et al. 1984; Gockenbach 1989), personality analyses indicated that mothers and fathers of gifted children were more assertive, with fathers also exhibiting greater liberalism and independence.

Parental personality plays a key role in shaping parental behavior and is considered a primary source of influence (Belsky 1984). A meta-analysis by Prinzie et al. (2009) found significant associations between parents’ personality, as assessed by the Five-Factor Model (McCrae and Costa 2010), and parenting practices. Higher levels of Extraversion, Agreeableness, Conscientiousness, and Openness, combined with lower levels of Neuroticism (McCrae and Costa 2010), were linked to warm, structured parenting characteristic of the authoritative style (Baumrind 1971, 1996). Such parents are better equipped to maintain positive interactions, respond to their child’s needs, and provide a coherent educational environment (Prinzie et al. 2009). Additionally, studies indicate that parents of gifted children and adolescents tend to be more flexible, more authoritative, and less authoritarian overall (Dwairy 2004; Pilarinos and Solomon 2017; Rudasill et al. 2013). Since parenting styles also appear to play a role in the cognitive development of children (Wang et al. 2022), it is not wrong to assume that parental personality might have a direct and indirect effect on the cognitive abilities of children.

In conclusion, although interest in parental personality characteristics has declined over time, the few positive findings that have emerged require systematic replication using up-to-date personality assessment tools.

### 1.5. The Present Study

The present study aimed to investigate the associations between parents’ cognitive abilities, educational background, and personality traits and the expression of gifted children’s cognitive abilities. The Wechsler Intelligence Scale for Children-Fourth Edition (WISC-IV) is the most widely used IQ test for assessing gifted children (Sparrow et al. 2005), and this study adopted this scale to assess cognitive ability in children.

#### 1.5.1. Parents’ Cognitive Ability and Children’s Intelligence

To examine the role of parents’ cognitive abilities on their children’s cognitive abilities, the present study focused on the extent to which the five CHC (Cattell–Horn–Carroll) factors in parents—Fluid Reasoning, Comprehension-Knowledge, Short-Term Memory, Visual Processing, and Processing Speed—obtained from the WAIS-IV are associated with the four primary indices in children (ICV, IRP, IML, IVE), as measured by the WISC-IV. We selected these five broad abilities for WAIS-IV because they provide more distinct measures of the underlying cognitive constructs (Lang et al. 2021). No norms for the same five broad CHC skills are available for the Italian edition of the WISC-IV.

Concerning the application of the CHC model and the Wechsler scales, some clarifications are needed: although the CHC model is the first psychometric model of cognitive abilities with a strong empirical basis, it is true that the latest Wechsler scales were not built according to the CHC model; on the contrary, the model was applied *a posteriori*. Over the course of time, both the classic and best-known four-factor structure (Verbal Comprehension, Perceptual Reasoning, Working Memory, and Processing Speed) and the five-factor structure (Comprehension-Knowledge, Fluid Reasoning, Visual Processing, Short-term Memory, Processing Speed) that corresponds to the CHC model (McGrew 1997) have been tested on the WAIS-IV Italian edition. The two models are complementary when used to derive WAIS-IV scores, offering clinicians the advantage of utilizing two psychometrically robust models for scoring the test and interpreting the results (Lang et al. 2021; Pezzuti et al. 2018). However, these models differ in several aspects (see below), prompting us to choose the CHC model, particularly regarding the decomposition of perceptual reasoning into separate Fluid Reasoning and Visual Processing abilities.

Verbal Comprehension Index (VCI; consisting of *Similarities*, *Vocabulary*, and *Information* subtests) and Comprehension-Knowledge (Gc; consisting instead of *Vocabulary* and *Information* subtests) can be considered on the whole as almost overlapping abilities and are both defined as the ability to comprehend and communicate culturally valued knowledge, encompassing both declarative and procedural knowledge, including language, vocabulary, and general knowledge acquired through learning and experience (Lang et al. 2021; Schneider and McGrew 2018). This ability is closely correlated with socioeconomic status (SES) and the quantity and quality of a person’s education (Lang et al. 2021).

As anticipated above, Fluid Reasoning (Gf) and Visual Processing (Gv) are treated as separate abilities within the CHC model, whereas the Wechsler scales combine them into the Perceptual Reasoning Index (PRI). Fluid Reasoning (Gf; consisting of *Matrix Reasoning* and *Figure Weights* subtests) refers to the ability to analyze novel and unfamiliar problems, identify underlying patterns and relationships, and apply logic to solve them (Schneider and McGrew 2018). It involves higher-order thinking processes such as concept formation, inference drawing, and reorganizing or transforming information, especially in situations that cannot be solved automatically (Otero 2017).

Visual Processing (Gv; consisting of *Block Design* and *Visual Puzzle* subtest) is the ability to perceive, analyze, synthesize, and manipulate visual patterns and stimuli (Schneider and McGrew 2018). It often involves solving problems using mental imagery of visual shapes and forms, commonly of a geometric or figural nature (Lang et al. 2021).

Short-Term Memory (Gsm) in the CHC model comprises the *Digit Span* and *Letter-Number Sequencing* subtests, while the Working Memory Index (WMI) in the Wechsler scales includes *Digit Span* and *Arithmetic* subtests. The latter, however, measures not only phonological memory but also quantitative reasoning. Short-Term Memory (Gsm) refers to the ability to apprehend, retain, and manipulate information within immediate awareness for a brief period—typically a few seconds. This limited-capacity system allows individuals to hold approximately seven chunks of information (plus or minus two) at a time before they fade from memory.

In contrast, the four-factor structure Processing Speed Index (PSI) and the broad ability Processing Speed (Gs) of the CHC model are superimposable. According to Schneider and McGrew (2018), Processing Speed (Gs) is the ability to control attention and automatically, quickly, and fluently perform simple, repetitive cognitive tasks. It is usually measured by tasks with fixed time intervals that require minimal mental effort or complex reasoning. In WAIS-IV both PSI and Gs consist of Coding and Symbol Search subtests.

Building on previous research (Anger and Heineck 2010; Grönqvist et al. 2010), we hypothesized that parental CHC cognitive abilities would be positively associated with the cognitive performance of their gifted children in the corresponding WISC-IV scores.

#### 1.5.2. Parents’ Education and Children’s Intelligence

Consistent with the literature (e.g., Cave et al. 2022; Cianci et al. 2013; Evans et al. 2010; Lemos et al. 2011; Rindermann and Baumeister 2015; Rindermann et al. 2010) and the findings from the Italian standardization sample of the WISC-IV (Pezzuti et al. 2019), we hypothesized that parental education would be significantly associated with the intellectual profiles of gifted children as measured by the WISC-IV. Specifically, we anticipate that gifted children whose parents have higher levels of education will achieve significantly higher WISC-IV scores than those whose parents have lower levels of education. This hypothesis reflects the established link between parental education and the provision of cognitively stimulating environments, as well as genetic predispositions for intellectual development.

#### 1.5.3. Parents’ Personality and Children’s Intelligence

To the best of our knowledge, no recent study has addressed the role of parental personality traits on the cognitive abilities of gifted children (e.g., Boyle 1988; Fell et al. 1984; Gockenbach 1989; Landau and Weissler 1993). Therefore, our hypotheses in this domain were highly exploratory. While we anticipated that personality traits in both fathers and mothers could be related to children’s cognitive performance, the specific traits and abilities involved were not predictable based on the existing literature. This lack of prior research underscores the need to examine these relationships systematically and to identify potential connections between parental personality and the various domains of children’s cognitive abilities.

## 2. Materials and Methods

### 2.1. Participants

This work is part of a larger ongoing research project that evaluates gifted children and their parents for several characteristics. It is based on data used for a preliminary descriptive analysis of cognitive abilities (Pezzuti et al. 2022), with additional data collected afterward and incorporated in new analyses. Specifically, we used a multivariate approach and updated previous findings by introducing new variables (parent education and personality traits). Participants were recruited through advertisements posted on the websites of parents’ associations for gifted children and relevant social media groups. Families voluntarily choose to participate without any form of compensation. As a result, we collected data from 65 gifted children and adolescents (21 girls and 44 boys) aged between 6 and 14 years (M = 9.91 years, SD = 2.24). The boys (M = 10.24 years, SD = 2.12) were older than the girls (M = 9.19 years, SD = 2.38), but this difference was not statistically significant (t(64) = 1.81, *p* = 0.075). Moreover, no differences were found between boys and girls regarding full-scale IQ. Where possible, both parents of each child were assessed, with data collected from 44 mothers and 41 fathers. Mothers’ ages ranged from 36 to 55 years (M = 44.13, SD = 4.07), while fathers’ ages ranged from 36 to 70 years (M = 45.90, SD = 6.13), t(40) = −2.67, *p* = 0.011, Cohen’s d = −0.42. Overall, mothers were more educated than fathers, t(40) = 4.89, *p* < 0.001, Cohen’s d = 0.76. No significant differences were found between mothers’ and fathers’ full-scale IQ (FSIQ) scores, t(36) = 1.68, *p* = 0.102, Cohen’s d = 0.28.

### 2.2. Procedures

Wechsler scales and personality self-report scales were administered specifically for this study, and data were not obtained via prior records. The time span between parents’ and children’s assessment never exceeded four weeks. In addition, we always ensured that an appropriate time interval (at least 12 months) elapsed since any previous administration of intelligence tests. Since all participants were administered the complete scales consisting of all core and supplementary subtests, the total duration of each administration was approximately 3 h. The administration was carried out by qualified clinicians.

Both children and parents were evaluated under standardized conditions at the psychological testing laboratory of the Department of Dynamic, Clinical, and Health Studies at Sapienza University of Rome. All participants voluntarily provided written informed consent prior to testing. The study received approval from the local ethical committee at the same department.

To confirm eligibility, children were required to achieve an FSIQ score of 120 or higher on the WISC-IV to qualify as gifted, in line with the most recent proposals from leading experts in giftedness (Ruf 2009; Silverman 2018), the latest research about gifted children (e.g., Zanetti et al. 2024). Although Carman (2013) noted that 54.2% of studies used an IQ of 130 as the cut-off for identifying gifted children and that a significant minority (27.6%) used a cut-off of 120, we chose to select for our sample all children with an FSIQ of 120 or higher. This decision aimed to avoid excluding gifted children from minorities or twice-exceptional gifted children, whose disabilities may lower their IQ scores (Silverman 2018). We also considered the standard error of measurement and the confidence interval; for example, a child with an IQ of 128 could have a score ranging from 123 to 135. Similarly, a student who scores 130 on the WISC-IV at one point in time could score between 125 and 135 on a subsequent retesting (Thompson and Morris 2008).

It is worth noting that, even if the GAI value would be chosen as the inclusion criterion, as recommended by some authors (Hagmann-von Arx et al. 2008; Rimm et al. 2008; Silverman 2009; Sparrow et al. 2005), our sample would have remained unaffected because all participants presented GAI values well above the cut-off of 120. The decision to use the IQ value as the sole criterion for inclusion stems from the fact that, in the absence of an unambiguous definition of giftedness, a large percentage of studies (62% of the studies conducted from 1995 to 2010 according to Carman 2013) use intelligence test scores as an identifying criterion.

### 2.3. Instruments

#### 2.3.1. Wechsler Intelligence Scale for Children-Fourth Edition-Italian Version (WISC-IV; Orsini et al. 2012)

The WISC-IV is a tool designed to assess the cognitive abilities of children and adolescents aged 6 to 16. It consists of 15 subtests—10 core and 5 supplementary—that yield the FSIQ, which is considered to reflect the general intelligence factor (g-factor). Additionally, the scale provides four primary scores corresponding to broad cognitive abilities: the Verbal Comprehension Index (VCI), Perceptual Reasoning Index (PRI), Working Memory Index (WMI), and Processing Speed Index (PSI). The average reliability coefficients across age groups in the Italian standardization sample were the following: VCI = 0.94; PRI = 0.92; WMI = 0.89; PSI = 0.84, IQ = 0.96 (see Orsini et al. 2012).

#### 2.3.2. Wechsler Adult Intelligence Scale-Fourth Edition-Fourth Edition-Italian Version (WAIS-IV; Orsini and Pezzuti 2013)

The WAIS-IV is a clinical tool used to assess cognitive abilities in individuals aged 16 to 90 years. Like the WISC-IV, it consists of 15 subtests (10 core and 5 supplementary) and provides the FSIQ and four primary scores: the Verbal Comprehension Index (VCI), Perceptual Reasoning Index (PRI), Working Memory Index (WMI), and Processing Speed Index (PSI). In addition, the WAIS-IV also evaluates five broad cognitive abilities based on the CHC model. The average reliability coefficients across age groups in the Italian standardization sample were the following: Gs = 0.92; Gc = 0.95; Gf = 0.93; Gv = 0.92; Gsm = 0.92 (see Lang et al. 2021).

#### 2.3.3. Big Five Inventory (BFI; John and Srivastava 1999; Ubbiali et al. 2013)

The BFI is a self-report questionnaire that assesses the Five-Factor Model of personality in individuals aged 18 and older. It measures five broad dimensions, which collectively describe emotional, interpersonal, experiential, attitudinal, and motivational styles. These are: Extraversion (E), which includes traits like assertiveness and social interaction; Agreeableness (A), covering trust, empathy, and altruism; Conscientiousness (C), reflecting duty, self-discipline, and orderliness; Neuroticism (N), ranging from emotional stability to anxiety and insecurity; and Openness to Experience (O), encompassing creativity, intellectual curiosity, and a preference for novelty. The BFI is widely used in both research and clinical settings for personality evaluation. The internal consistency for the Italian sample is as follows: E = 0.80, A = 0.69, C = 0.83, N = 0.80, O = 0.80 (Ubbiali et al. 2013).

### 2.4. Data Analysis

#### 2.4.1. Assumptions

Before conducting the main analyses, we examined missing data, normality assumptions, multicollinearity, and data clustering. Following our analysis plan, we conducted separate missing-value analyses and multicollinearity diagnostics for two variable sets: Set A (children’s and parents’ cognitive abilities, parents’ education) and Set B (children’s cognitive abilities, parents’ personality traits, parents’ education). Up to 14% of data were missing (nine cases) for mothers’ and fathers’ Gf. Little’s MCAR test was not significant for Set A, indicating that the data were missing completely at random, but was marginally significant (*p* = 0.016) for Set B, suggesting that those missing values may be missing at random. Given the relatively small sample size, we used the Expectation-Maximization algorithm in SPSS 27 to impute missing data. However, caution is advised when interpreting analyses for Set B, where the MCAR assumption does not fully hold.

After imputation, we assessed the normality of variables in Table 1. Although Shapiro–Wilk tests were significant for several variables, most had W values above 0.95 (close to perfect normality). Four variables had W values slightly below 0.95 (mothers’ education, Openness; fathers’ Gs, Conscientiousness), and only fathers’ education showed a W value near 0.90. However, skewness and kurtosis values for all variables remained within ±2 (Tabachnick et al. 2018), indicating only minor deviations from normality. Consequently, we added non-parametric analyses to gauge the influence of non-normality on parametric results.

The primary hypotheses of the study were tested using PLS regression models (see Statistical Analysis), which require that multicollinearity among predictors be minimized. Multicollinearity was assessed on mean-centered variables (see analysis plan below) using the *metan* package for R (Olivoto and Lúcio 2020). The results indicated weak multicollinearity for both sets. For the first set, the condition numbers (CNs), calculated as the ratio between the largest and smallest eigenvalues of the correlation matrices for the dependent and independent variables, were 2.60 for children’s cognitive abilities and 19.79 for parents’ cognitive variables. For the second set, the CNs were 2.60 for children’s cognitive abilities and 9.28 for parents’ personality variables. Collinearity between children’s and parents’ variables was intentionally disregarded, as the substantive research hypotheses focused on examining the contribution of parental variables to explaining children’s WISC-IV outcomes. CN values below 10 suggest no multicollinearity, values between 10 and 30 indicate some concern, and values above 30 signify substantial multicollinearity. Additionally, none of the variance inflation factors (VIFs) exceeded 10, and no correlations were above |0.80|, further confirming the absence of multicollinearity issues.

Given that 17 mothers and 18 fathers contributed multiple children, we considered potential clustering (i.e., sibling similarity) in the data. Clustering can introduce bias if treated as independent observations. We used the Intraclass Correlation Coefficient (ICC1) to estimate the proportion of variance due to family-level differences. For mother–child dyads, ICC1 values were VCI (0.19), PRI (0.31), WMI (−0.46), and PSI (0.50); for father–child dyads, ICC1 values were VCI (0.09), PRI (0.36), WMI (−0.44), and PSI (0.49). According to recommended cut-offs, ICC1 < 0.05 indicates negligible clustering, 0.05–0.20 modest group effects, and >0.20 substantial clustering. Although some values indicated moderate to substantial clustering (e.g., PSI, PRI), hierarchical modeling was infeasible due to the small, non-normally distributed sample. Instead, we used Partial Least Squares (PLS) regression, which is well-suited for exploratory analysis in smaller samples but lacks hierarchical capabilities. Clustering effects were addressed through resampling (e.g., bootstrapping) within the PLS framework.

#### 2.4.2. PLS Regressions

General Strategy. We used Partial Least Squares Structural Equation Modeling (PLS-SEM) in SmartPLS 4 for its suitability with non-normal data and small samples (Hair et al. 2017; Iranmanesh et al. 2024). Several models examined relationships between children’s and parents’ cognitive abilities and between children’s cognitive abilities and parents’ personality traits, controlling for education and biological sex.

Analysis Plan. We first carried out basic analyses in which we tested parents’ variables (cognitive abilities or personality traits) on children’s cognitive outcomes—PRI, VCI, PSI, and WMI. Predictors were entered in steps: mothers’ and fathers’ variables (Model 1a), then parents’ education (Model 1b). Next, we included children’s biological sex as a moderator of these main effects (Model 2), adding interaction terms and mean-centering predictors (Aiken et al. 1991). Using findings from Models 1 and 2 and the intercorrelation matrix, we tested multivariate models with significant predictors. Model 3a examined parental education and cognitive abilities on children’s PRI, VCI, PSI, and WMI; Model 3b examined parental education and personality traits on the same outcomes. Finally, Model 4 integrated education, cognitive abilities, and personality traits into one framework to evaluate independent and combined effects on children’s cognitive abilities.

Model Comparisons. We identified best-fitting models by comparing R^2^, Q^2^, SRMR, and BIC (Danks et al. 2020; Hair et al. 2017; Shmueli et al. 2019). R^2^ measures variance explained (≥0.75 = substantial, ≥0.50 = moderate, ~0.25 = weak). Q^2^ > 0 indicates predictive relevance (0.02 = small, 0.15 = medium, 0.35 = large). SRMR < 0.08 suggests a good fit. BIC balances fit with complexity (lower BIC = better).

Tests of Regression Coefficients. SmartPLS uses bootstrapping (10,000 replications) with bias-corrected confidence intervals (90% CI) to assess significance. This approach is well suited for non-normal and small samples, producing robust estimates by resampling the data.

## 3. Results

Table 2 reports the bivariate correlations between children’s WISC-IV scores with mothers’ and fathers’ education and CHC broad abilities using parametric and non-parametric methods (below and above the diagonal, respectively).

The relationships among maternal CHC abilities, education, and children’s cognitive performance on the WISC-IV showed several significant associations. Regarding the children’s VCI, we found a moderate, positive correlation with maternal education (r = 0.37, *p* < 0.01). This suggests that children who performed better on the VCI had mothers with higher levels of education. Additionally, children’s VCI was also associated with maternal Fluid Intelligence (r = 0.21, *p* = 0.05). Regarding the children’s PRI, we found modest positive correlations with maternal education (r = 0.23, *p* < 0.05) and Visual Processing ability (r = 0.29, *p* < 0.05). However, the correlation between maternal education and the children’s PRI did not reach statistical significance when analyzed using the Spearman method (r = 0.17, *p* = 0.091). The children’s WMI was not associated with the mothers’ CHC abilities. However, the children’s PSI was positively correlated with maternal Processing Speed (r = 0.29, *p* < 0.01) and, unexpectedly, with maternal Comprehension-Knowledge ability (r = 0.22, *p* < 0.05). While the former association was also detected using the non-parametric Spearman method (r = 0.28, *p* < 0.05), the latter was no longer significant (r = 0.19). The Spearman method also indicated a modest positive correlation between the children’s PSI and maternal Visual Processing ability (r = 0.21, *p* < 0.05).

The relationship between paternal CHC abilities, education, and children’s cognitive performance on the WISC-IV resulted only in a positive correlation between the children’s WMI and fathers’ short-term memory (r = 0.30; *p* < 0.001), suggesting that paternal memory span could be linked to children’s learning abilities.

The bivariate correlations between children’s WISC-IV scores and parents’ education and personality traits are fully reported in Supplementary Table S1. Here, we present the key findings, which were somewhat modest. From an inspection of the correlation matrix, we found only two statistically significant correlations: maternal Conscientiousness with the PRI (r = 0.36, *p* < 0.01 and r = 0.39, *p* < 0.01 using the Pearson and Spearman methods, respectively) and paternal Agreeableness with the PRI (r = 0.29, *p* < 0.05 using both methods).

To determine the best-fitting PLS model, we evaluated R^2^, SRMR, Q^2^, and BIC (see methods), as reported in Table 3. For VCI prediction, Model 1b demonstrated a good balance of fit, with a moderate R^2^, a positive Q^2^, and the lowest BIC, making it the best choice. Model 2, while explaining more variance, had a negative Q^2^, indicating poor predictive relevance, and a higher BIC, suggesting a less favorable fit. Despite its poorer fit, we also examined Model 2 coefficients, as it explored interaction terms between child biological sex and parental cognitive abilities. For WMI, Model 1a outperformed Model 2, with a higher SRMR, a positive Q^2^, and the lowest BIC, making it the preferred model, despite its relatively lower R^2^. Model 2 showed a higher R^2^ but a negative Q^2^, signaling poor predictive relevance. As with VCI, we also inspected the coefficients of Model 2 for possible interactions. For PRI, Model 1b provided the best fit, with moderate explained variance (R^2^) and positive predictive relevance, while Model 2 had a higher R^2^ but a negative Q^2^, indicating weak predictive relevance. Finally, for PSI, Model 1a was the best model, with a positive Q^2^ and the lowest BIC, compared to Model 2’s higher R^2^ but negative Q^2^, signaling poor predictive relevance. In summary, Models 1b and 1a consistently demonstrated better overall fit, making them the optimal choices for the dependent variables.

Table 4 reports the bootstrap tests of model coefficients. For PSI, maternal Gs consistently emerged as a significant predictor (B = 0.30) across Models 1a and 1b. In Model 2, maternal Gs remained significant (B = 0.28), while interactions between children’s biological sex and parental variables were not significant. Paternal Gs was not a significant predictor across all models. For WMI, Model 1a indicated a significant effect of paternal Gsm (B = 0.24), whereas maternal Gsm did not achieve statistical significance. In Model 1b, none of the predictors were significant, confirming the worst fit relatively to Model 1a. In Model 2, paternal Gsm remained a significant predictor (B = 0.38), and the only significant interaction observed was between children’s biological sex and maternal Gsm (B = −0.74). The simple slope analysis showed that maternal Gsm was a stronger predictor of children’s WMI in females compared to males. For PRI, maternal Gf emerged as a significant predictor in Model 1a (B = 0.20), whereas paternal Gf was not significant. When PRI was predicted by parental Gv, paternal Gv was the only significant predictor in Model 1a (B = 0.20). In Model 1b, which addressed parental education, suppression effects were observed: maternal Gf was no longer significant, and paternal Gv became a negative predictor of PRI (B = −0.21). Model 1b showed a poorer fit compared to Model 1a. In model 2, no moderation effect was found. In predicting VCI, neither maternal nor paternal Gc showed statistically significant associations with children’s scores across all models. Model 1b indicated a significant effect of maternal education (B = 0.38), while paternal education did not contribute substantially. In Model 2, we found an interaction between children’s biological sex and maternal education (B = 0.69). Again, the simple slope analysis showed that maternal education was a stronger predictor of children’s VCI in males compared to females. In sum, maternal CHC abilities and education generally had stronger predictive power compared to paternal ones, especially for verbal and cognitive processing speed. Parental abilities were identified as significant predictors of children’s working memory and perceptual reasoning.

Consistent with the bivariate correlation analysis, the analyses using parental personality traits as predictors of children’s performance on the WISC-IV yielded relatively modest results. As shown by the models’ fit indices (fully reported in Supplementary Table S2), no model with PSI as the dependent variable proved acceptable, either in terms of explained variance (R^2^) or predictive ability, in cross-validation analyses (Q^2^). For the VCI, the models (1b) using parental personality traits as predictors, while controlling for the educational level of both parents, were acceptable across all fit indices. However, significance tests for the regression coefficients (Supplementary Tables S3–S7) indicated that the models’ performance was largely driven by the significant effect of parental education, with the only novel finding being the association of maternal Agreeableness with children’s VCI (B = 0.19) (Supplementary Table S4). For the WMI, the only acceptable model (across all fit indices) was the one using only the Agreeableness of both parents to predict children’s WISC-IV scores. Specifically, paternal Agreeableness was statistically significant (B = 0.20) in the regression coefficient tests (Supplementary Table S4). This finding was not found at the level of bivariate correlations. Finally, PRI was predicted by maternal Conscientiousness (B = 0.32) (Supplementary Table S5). This finding was already evident at the level of bivariate correlations.

After exploring predictive models separately for each dependent variable (VCI, WMI, PRI, and PSI), we proceeded to assess the performance of different sets of predictors within a unified predictive model. Specifically, we tested Model 3a, which incorporated the cognitive abilities identified as the best predictors in Models 1 and 2 to predict VCI, WMI, PRI, and PSI within a single framework. Similarly, Model 3b included personality traits that emerged as top predictors in Models 1 and 2, also applied to the combined dependent variables. Finally, our last model, named Model 4, integrated the predictors from both Model 3a and Model 3b to create a comprehensive multivariate framework.

**Figure 1:**
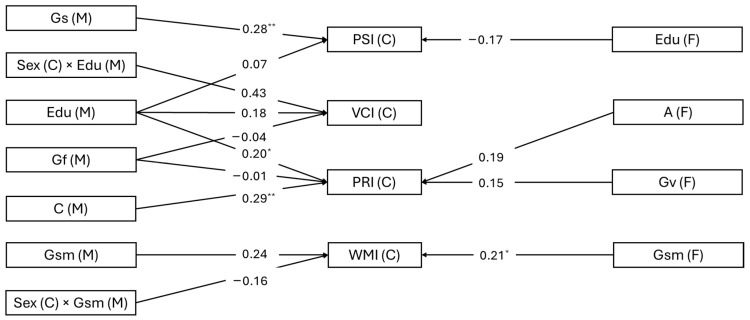
Results of path analysis Model 4. Mothers’ and fathers’ CHC abilities, personality, and education were used to predict children’s WISC-IV scores simultaneously. Path coefficients are standardized regression coefficients. Bootstrap significance levels are indicated with asterisks: * *p* < 0.05; ** *p* < 0.01; (one-tailed).

To determine the best-fitting PLS model, we evaluated R^2^, SRMR, Q^2^, and BIC, for each dependent variable (VCI, WMI, PRI, and PSI) as reported in Table 5. For VCI prediction, Models 3a and 4 displayed identical R^2^, Q^2^, and BIC values, indicating no differences in predictive relevance or parsimony. However, Model 4 had a slightly better SRMR, suggesting a marginally superior fit. Regarding WMI, Model 4 and Model 3a showed identical R^2^, while Model 3b showed negligible explanatory power. The other fit indices (SRMR, Q^2^, and BIC) were nearly equivalent for Models 4 and 3a, making Model 4 slightly preferable due to its slightly lower SRMR while maintaining similar predictive relevance and parsimony. For PRI, Model 4 exhibited the highest R^2^, followed by Model 3b. Although Model 4 had the lowest SRMR (0.074), Model 3b was superior in terms of predictive relevance and parsimony, as evidenced by Q^2^ and BIC. The decision between Models 3b and 4 depended on the priority given to predictive relevance (favoring Model 3b) or explained variance and fit (favoring Model 4). Lastly, for PSI, Models 4 and 3a explained the same variance, with identical Q^2^ and BIC values. However, Model 4 had a slightly lower SRMR, leading us to conclude that it was marginally better overall. Taken together, we opted for Model 4 to assess the association of parents’ education, intelligence, and personality with their children’s cognitive ability simultaneously (Models 3a and 3b are reported in Supplementary Figure S1). In Figure 1, we have presented the path coefficients of Model 4.

For PSI and WMI, the bootstrap significance test indicated that both WISC-IV indices were associated with the corresponding parental abilities. However, while PSI was significantly associated with the mothers’ Gs, WMI was significantly associated with the fathers’ Gsm. The relationship between the mothers’ Gsm and the child’s WMI was of moderate magnitude, but unfortunately, the bootstrap test did not support the statistical significance of this path. The child’s PRI was significantly associated with maternal education and maternal conscientiousness. Regarding VCI, Model 4 refuted the significant relationships of this WISC-IV index with maternal education and the interaction between maternal education and the child’s biological sex. These path coefficients were smaller in magnitude compared to the estimates obtained in Model 2 and did not reach statistical significance.

## 4. Discussion

The present study explored the role of parental education, cognitive abilities, and personality traits in shaping the cognitive abilities of gifted children. Drawing upon the CHC model (McGrew 1997) and the Five-Factor Model of personality (McCrae and Costa 2010), we hypothesized that parental characteristics would predict their children’s performance on the WISC-IV. Specifically, we expected that parental CHC abilities would align with corresponding WISC-IV indices in children (Anger and Heineck 2010; Grönqvist et al. 2010; Pezzuti et al. 2022), parental education would contribute to their children’s overall cognitive performance (Cave et al. 2022; Cianci et al. 2013; Pezzuti et al. 2019; Rindermann and Ceci 2018), and parental personality traits would exhibit domain-specific associations. In the following paragraphs, we comment on how our findings supported each specific hypothesis, comparing simple bivariate correlations with multivariate regression analyses.

### 4.1. Parents’ Cognitive Ability and Children’s Intelligence

The hypothesis that parental CHC abilities would predict corresponding cognitive indices in children was partially supported. Bivariate correlations indicated significant associations between parental cognitive abilities and children’s performance. For mothers, significant correlations were observed between Gf and children’s VCI, Gv and children’s PRI, and both Gs and Gc with children’s PSI. For fathers, only Gsm showed a significant correlation with children’s WMI. These findings align with previous studies, such as Min et al. (2007), who identified relationships between mothers’ processing speed and children’s cognitive processing, and Anger and Heineck (2010), who found similar associations for coding speed. However, some findings were unexpected, such as the correlations of maternal Gc and Gf with children’s PSI and VCI, respectively. When the data were analyzed using PLS-SEM, which accounted for multivariate interactions and controlled for parental education, a more nuanced pattern emerged.

First, despite a significant bivariate correlation of children’s VCI with mothers’ Gf and Education, no parental cognitive variables significantly predicted VCI in the multivariate analyses. This finding indicated that the initial associations may be confounded by other parental factors. Interestingly, maternal education appeared to be a stronger predictor of children’s VCI in boys compared to girls. Yet, when this interaction was included in the multivariate models, the direct effect of maternal education on VCI became nonsignificant. Second, although bivariate correlations indicated that mothers’ Gv was related to children’s PRI, this effect became less robust in the multivariate analyses. Specifically, the inclusion of parental education altered the weight of Gv on PRI, suggesting that the observed associations may be spurious or mediated by educational factors. This finding highlighted the importance of accounting for educational background of the family when studying the inheritance of cognitive abilities.

In addition to somewhat unexpected findings, which required confirmation in future studies, our research also identified very consistent patterns, which remained statistically significant after controlling parental education and other cognitive abilities. These findings, not surprisingly, align with previous research. First, mothers’ Gs consistently emerged as the strongest predictor of children’s PSI across all models. This robust finding is consistent with Min et al. (2007) and Anger and Heineck (2010), as well as with the broader understanding of the role of maternal processing speed in shaping children’s cognitive efficiency. Second, fathers’ Gsm emerged as the best predictor of children’s WMI across bivariate and multivariate analyses. This result corroborated findings by Min et al. (2007), who observed similar patterns using digit span tests, and aligns with theoretical perspectives on the intergenerational transmission of short-term memory abilities.

These results suggest domain specificity in the relation of parental cognitive abilities with children’s performance. Maternal Gs played a central role in predicting children’s cognitive speed (PSI), possibly reflecting the direct inheritance of processing efficiency or the influence of a cognitively stimulating environment. Meanwhile, fathers’ Gsm had a stronger impact on children’s working memory (WMI), aligning with the heritability of short-term memory abilities. These findings also underscore the importance of considering both genetic and environmental factors, as highlighted by McGuffin and Scourfield (1997), who noted that identical genes may manifest differently depending on whether they are inherited maternally or paternally.

In summary, while bivariate correlations provided initial insights into parent–child cognitive relationships, PLS-SEM highlighted the complexity of these dynamics, revealing interactions and confounding effects that bivariate methods may overlook. This approach emphasized the importance of maternal processing speed and paternal short-term memory as key predictors of children’s cognitive abilities, reinforcing the need for a domain-specific understanding of parental influences in the study of giftedness. However, it is worth noting that, while specific cognitive abilities may be similar between parents and children, genetic influences can manifest in different phenotypes depending on whether they are inherited from the mother or the father (McGuffin and Scourfield 1997). Furthermore, it is worth noting that parental characteristics appear to predict cognitive abilities—such as WMI and PSI—that are less prominent in defining giftedness and often represent areas of relative weakness for gifted children (Morrone et al. 2019; Pezzuti et al. 2022).

### 4.2. Parental Education and Children Intelligence

The bivariate correlations showed that the mothers’ level of education was associated with both VCI and PRI. Using single PLS-SEM models, we found that mothers’ education only predicted children’s VCI. Finally, analyzing the multivariate model, in which all variables found to be statistically significant in the previous analyses were included, it emerged that mothers’ education predicted children’s PRI, a result that was already evident in the bivariate correlations. The idea that maternal education influences children’s cognitive skills, particularly in Fluid Reasoning and visuo-perceptual domains, is not novel. A seminal study by Mercy and Steelman (1982) demonstrated that maternal education directly impacted performance on the Wechsler Block Design subtest. In other studies (Brooks 2011; Cave et al. 2022; Cianci et al. 2013; Craig 2006; Duncan and Magnuson 2012; Meekes et al. 2015; Mercy and Steelman 1982; Rindermann and Baumeister 2015) it emerged that maternal education rather than paternal education influenced children’s abilities, particularly the verbal domains. It is well-documented that parents from highly educated backgrounds foster learning through high-quality, child-directed language (Rowe 2012). Educated parents are more likely to provide enriching childcare resources, including books, learning materials, and educational activities (Davis-Kean 2005; Raviv et al. 2004). Our finding highlights the possible impact of maternal education on children’s Fluid Reasoning, because mothers often serve as primary caregivers and spend substantial time with their children (Carneiro et al. 2013; Harding 2015; Mercy and Steelman 1982), they have a considerable influence on their upbringing. However, fathers have increasingly become more involved in childcare, particularly in recent generations.

### 4.3. Parental Personality and Children’s Intelligence

The hypothesis that parental personality traits would be linked with children’s cognitive abilities was partially supported, revealing weaker and less consistent effects compared to cognitive abilities and education. It is worth noting that our hypotheses in this domain were highly exploratory, given that the relationship between parental personality and children’s cognitive abilities remains understudied. Nonetheless, maternal Conscientiousness emerged as a significant predictor of children’s Perceptual Reasoning Index (PRI) in all analyses, suggesting that structured and organized parenting could enhance visual-spatial reasoning skills. Some evidence supports the idea that conscientious parents often create disciplined and task-focused environments conducive to developing specific cognitive abilities, such as perceptual reasoning. For example, Bornstein et al. (2011) indicated that conscientious mothers could be more likely to actively engage with their children, demonstrate increased sensitivity, and perceive themselves as effective and capable in their parenting roles. In keeping with this view, mothers of gifted children were also found to be high in conscientiousness-related personality characteristics and perseverance (e.g., Boyle 1988; Gockenbach 1989; Fell et al. 1984), but no study so far has found a direct correlation between maternal Conscientiousness and performance in cognitive tasks requiring fluid and visual abilities. Another finding of our study was the association of paternal agreeableness with children’s PRI in the bivariate analysis, suggesting that paternal traits associated with empathy, cooperation, and positive interpersonal interactions may contribute to creating a cognitively supportive environment. However, this relationship did not remain significant in the multivariate models, indicating that the observed effect may have been confounded by other parental characteristics, such as education. Overall, our findings suggest that, while personality traits may correlate with the cognitive environment in which children develop, their effects are likely overshadowed by the more direct contributions of parental cognitive abilities and education. This is consistent with the broader understanding that personality traits primarily shape parenting behaviors and the emotional climate of the household, which may have indirect effects on cognitive development.

### 4.4. Limitations and Future Directions

Inevitably, the current study has noteworthy limitations that, while necessitating caution in interpreting the findings, provide valuable insights for future research directions. First, a notable limitation of this study was the presence of moderate intraclass correlation coefficients (ICC1) for certain variables (e.g., PRI and PSI), indicating non-negligible clustering effects. However, the use of Partial Least Squares (PLS) regression, necessitated by the small and non-normally distributed sample, precluded the implementation of multilevel modeling, which may have provided a more nuanced understanding of the hierarchical data structure. These methodological decisions, though appropriate for the data constraints, should be considered when interpreting the results, particularly regarding the role of family dynamics on the observed relationships. Results involving PSI, while potentially subject to clustering bias, are among the most reported findings in similar studies (Min et al. 2007; Anger and Heineck 2010), lending them a certain robustness in interpretation. In contrast, the results for PRI, although also potentially affected by clustering bias, did not emerge as fully significant; in this case, disregarding clustering tends to inflate Type I errors rather than diminish them. It is evident that future studies involving families with more than one gifted child will need to consider hierarchical models. However, such approaches require large sample sizes to ensure reliable and precise estimates.

Other limitations relate to the sample size, the biological sex imbalance, the wide age range of the assessed children and adolescents (6–14 years), and the lack of a control group. While the latter issue prevented us from determining whether parental education, cognitive abilities, and personality traits have different weights in influencing intelligence in gifted children compared to typically developing peers, the former factors limited our ability to analyze whether and how parental characteristics could be associated with children’s cognitive abilities across developmental stages, making it difficult to address the nature vs. nurture debate. It is well established that the contribution of shared environmental factors decreases, while genetic influence increases, with age from childhood to adolescence (Deary et al. 2006). Expanding the sample size, adding a control group, and conducting studies within narrower age ranges will be crucial. However, as noted by the NAGC (2025), gifted individuals constitute only the top 10% of the population, which poses inherent recruitment challenges.

Additionally, our study focused on personality factors, leaving other critical aspects within the child’s microsystem underexplored (e.g., Bronfenbrenner 1986). The Munich Model of Giftedness (Heller 2004; Heller et al. 2005) underscores the importance of family climate, family functioning, and parenting style, as well as school-related factors like quality of instruction and classroom climate, in moderating the expression of giftedness. Future research should incorporate assessments of these broader contextual influences to provide a more holistic understanding of the factors shaping gifted children’s cognitive abilities.

Further, alternative or supplementary methods for identifying giftedness—such as qualitative judgments, curriculum-based measures, and creativity tests (e.g., Cao et al. 2017)—might yield different results. Exploring these methods would enhance our understanding of giftedness and its diverse manifestations. Relying solely on IQ results may cause some gifted populations to be overlooked since IQ tests are not as helpful in identifying someone with creative, leadership, or other abilities; however, in Italy, the exclusive use of intelligence tests is the most common strategy for identifying gifted individuals. Although we have amply justified our choice of using the cut-off of an IQ score of 120 for recruiting the gifted sample, in the light of the still unresolved debate on what is the best cut-off for identifying gifted subjects, this choice also brings with it limitations, since, once again, it could limit the generalizability of the data obtained.

Finally, longitudinal studies are vital for validating causal pathways and disentangling the complex interplay between genetic and environmental factors in the cognitive development of gifted children.

## 5. Conclusions

By comparing bivariate and multivariate approaches, the current study highlighted the value of advanced statistical methods in capturing the complex interplay of parental intelligence, education, and personality in shaping children’s giftedness. The differential association of mothers’ and fathers’ characteristics with children’s cognitive abilities offers insights into the genetic and environmental underpinnings of cognitive performance. However, further research is required to clarify the mechanisms behind these differing associations across cognitive domains. As discussed, the results were not entirely as anticipated, and some inconsistencies emerged across models. These findings suggest the need for deeper investigation into how parental characteristics influence the specific subtests that constitute the WISC-IV indices, as well as other measures of intelligence. Future studies could explore whether these effects vary across different assessments or contexts. Our findings contribute to the existing literature on giftedness and underscore the importance of comprehensive approaches to understanding and fostering intellectual potential in children. By incorporating genetic, cognitive, and environmental factors, future research can provide a more comprehensive framework for supporting gifted individuals.

## Figures and Tables

**Table 1:** Descriptive statistics and normality tests for children’s cognitive variables and parents’ cognitive and personality variables.

**Children Variables**								
	**Mean**	**SD**	**Min**	**Max**	**Skewness**	**Kurtosis**	**Shapiro–Wilk (W)**	***p***
VCI	131.85	9.39	112	152	−0.11	−0.70	0.98	0.416
PRI	134.15	10.74	113	154	0.01	−0.91	0.97	0.107
WMI	115.55	11.06	94	145	0.39	−0.20	0.98	0.206
PSI	112.73	13.70	82	150	0.11	0.01	0.99	0.717
**Mothers’ Variables**								
	**Mean**	**SD**	**Min**	**Max**	**Skewness**	**Kurtosis**	**Shapiro–Wilk (W)**	***p***
Education	17.77	3.05	13	25	0.28	−0.18	0.94	0.005
Gs	119.97	13.92	89	150	−0.35	−0.66	0.96	0.035
Gc	119.31	10.39	100	139	0.10	−0.90	0.97	0.071
Gf	120.16	13.50	91.77	143	−0.32	−0.84	0.96	0.032
Gv	117.68	13.06	86	156.85	−0.00	0.47	0.96	0.034
Gsm	111.02	9.44	93.49	136	0.22	−0.17	0.98	0.203
E	49.83	11.51	21	69	−0.43	−0.65	0.96	0.025
A	49.45	9.63	28	68	−0.54	−0.04	0.96	0.022
C	52.73	7.31	39	67	0.13	−0.83	0.97	0.125
N	49.60	9.91	27	72	0.21	0.25	0.96	0.033
O	55.85	7.44	37	68	−0.59	−0.59	0.92	0.001
**Fathers’ variables**								
	**Mean**	**SD**	**Min**	**Max**	**Skewness**	**Kurtosis**	**Shapiro–Wilk (W)**	***p***
Education	15.11	3.14	8	21	−0.53	−0.13	0.89	0.001
Gs	114.25	11.60	95	133	−0.18	−1.06	0.94	0.003
Gc	117.07	15.16	81	147	−0.42	−0.04	0.96	0.031
Gf	119.70	11.96	94	138.31	−0.34	−0.73	0.96	0.024
Gv	116.35	13.59	86	149	0.46	−0.06	0.97	0.109
Gsm	111.90	13.82	78	147	−0.16	0.33	0.96	0.053
E	50.23	9.44	31	66	−0.33	−0.76	0.95	0.010
A	48.06	10.48	26	66	−0.44	−0.77	0.95	0.010
C	56.06	9.03	29	69	−0.73	0.40	0.95	0.007
N	43.14	9.32	25	65.34	0.21	−0.40	0.98	0.329
O	53.40	9.22	33	70.67	−0.35	−0.49	0.96	0.037

Legend. Gs = Processing Speed, Gc = Comprehension-Knowledge, Gf = Fluid Reasoning, Gv = Visual Processing, Gsm = Short-Term Memory, E = Energy, A = Agreeableness, C = Conscientiousness, N = Neuroticism, O = Openness, SD = Standard Deviation.

**Table 2:** Correlation matrix of children WISC-IV scores with mothers’ and fathers’ education and CHC broad abilities using Pearson (parametric) and Spearman (non-parametric) methods.

	1.	2.	3.	4.	5.	6.	7.	8.	9.	10.	11.	12.	13.	14.	15.	16.
1. VCI (C)	--	0.17	−0.12	−0.12	0.36 **	−0.11	0.15	0.22 *	0.02	−0.06	0.09	−0.05	0.06	−0.06	−0.07	−0.12
2. PRI (C)	0.18	--	0.33 **	0.18	0.17	0.15	0.05	0.16	0.27 *	0.15	0.11	0.00	−0.06	−0.10	0.15	0.00
3. WMI (C)	−0.12	0.32 **	--	0.22 *	−0.03	−0.14	0.06	0.03	0.15	0.12	0.13	0.05	0.04	0.07	0.05	0.27 *
4. PSI (C)	−0.13	0.17	0.26 *	--	−0.02	0.28 *	0.19	0.03	0.21 *	−0.10	−0.17	−0.04	0.02	0.03	0.03	−0.08
5. Edu (M)	0.37 **	0.23 *	−0.02	0.01	--	−0.02	0.41 **	0.43 **	0.19	0.00	0.24 *	0.24 *	0.07	0.19	−0.10	−0.18
6. Gs (M)	−0.06	0.18	−0.18	0.29 **	0.05	--	−0.06	0.12	0.16	0.14	−0.06	0.34 **	0.49 **	0.16	0.42 **	0.03
7. Gc (M)	0.14	0.04	0.06	0.22 *	0.39 **	−0.03	--	0.36 **	0.18	0.07	0.15	0.15	0.10	0.20	0.02	−0.24 *
8. Gf (M)	0.21 *	0.13	0.00	0.00	0.45 **	0.19	0.32 **	--	0.03	0.35 **	0.08	0.06	0.08	0.10	0.00	0.02
9. Gv (M)	0.04	0.29 **	0.15	0.19	0.22 *	0.17	0.21 *	−0.01	--	0.16	0.09	0.206 *	0.07	0.14	0.01	0.12
10. Gsm (M)	−0.07	0.15	0.08	−0.16	0.04	0.19	0.10	0.40 **	0.23 *	--	−0.22 *	0.10	−0.03	−0.13	0.17	0.13
11. Edu (F)	0.09	0.12	0.14	−0.18	0.26 *	−0.02	0.17	0.07	0.10	−0.23	--	0.18	0.50 **	0.57 **	0.26 *	0.25 *
12. Gs (F)	−0.03	0.00	0.02	−0.03	0.24 *	0.39 **	0.18	0.06	0.23 *	0.07	0.29 *	--	0.50 **	0.57 **	0.37 **	0.24 *
13. Gc (F)	0.11	−0.04	0.02	−0.01	0.18	0.45 **	0.14	0.06	0.10	−0.03	0.58 **	0.55 **	--	0.66 **	0.46 **	0.15
14. Gf (F)	−0.07	−0.10	0.09	−0.01	0.21 *	0.15	0.19	0.13	0.08	−0.13	0.61 **	0.55 **	0.62 **	--	0.31 **	0.37 **
15. Gv (F)	−0.08	0.20	0.06	0.03	0.06	0.41 **	0.06	0.02	0.20	0.17	0.30 **	0.36 **	0.39 **	0.37 **	--	0.05
16. Gsm (F)	−0.06	0.04	0.30 **	−0.10	−0.12	0.01	−0.22 *	0.10	0.11	0.13	0.33 **	0.32 **	0.21 *	0.46 **	0.13	--

** Correlation is significant at the 0.01 level (one-tailed). * Correlation is significant at the 0.05 level (one-tailed). Note: Pearson and Spearman correlations are below and above the diagonal, respectively. Legend: VCI = Verbal Comprehension Index; PRI = Perceptual Reasoning Index; WMI = Working Memory Index; PSI = Processing Speed Index; Gs = Processing Speed; Gc = Comprehension-Knowledge; Gf = Fluid Reasoning; Gv = Visual Processing; Gsm = Short-Term Memory; (C) = children variables; (M) = mother variables; (F) = father variables.

**Table 3:** PLS regressions. Model fit for basic and moderation analyses. Dependent variables are children’s WISC-IV scores, and predictors are parental CHC abilities. Each child’s score is predicted by the corresponding CHC ability.

**(a) Dependent: VCI**					**(b) Dependent: WMI**				
Model	R^2^	SRMR	Q^2^	BIC	Model	R^2^	SRMR	Q^2^	BIC
1a	0.02	0.00 ^†^	−0.04	10.25	1a	0.10	0.00 ^†^	0.04	4.93
1b	0.14	0.00 ^†^	0.03	9.75	1b	0.11	0.00 ^†^	−0.02	12.64
2	0.28	0.08	−0.04	19.59	2	0.20	0.04	−0.05	26.41
**(c) Dependent: PRI ^‡^**					**(d) Dependent: PSI**				
Model	R^2^	SRMR	Q^2^	BIC	Model	R^2^	SRMR	Q^2^	BIC
1a	0.04 (0.08)	0.00 ^†^ (0.00 ^†^)	−0.02 (0.03)	8.91 (6.23)	1a	0.08	0.00 ^†^	0.03	5.93
1b	0.09 (0.11)	0.00 ^†^ (0.00 ^†^)	−0.04 (0.04)	13.74 (12.43)	1b	0.10	0.00 ^†^	0.00	12.76
2	0.17 (0.17)	0.06 (0.07)	−0.21 (−0.03)	28.44 (28.91)	2	0.23	0.08	−0.10	23.89

Legend: VCI = Verbal Comprehension Index; PRI = Perceptual Reasoning Index; WMI = Working Memory Index; PSI = Processing Speed Index; Note: ^†^ = Saturated model has SRMR equal to 0.00; ^‡^ = For PRI analyses the values in parentheses indicate that the predictor is Gv, while those outside the parentheses indicate that the predictor is Gf.

**Table 4:** PLS regressions. Model fit for basic and moderation analyses. Dependent variables are children WISC-IV scores, predictors are parental broad abilities and education. Each child’s score is predicted by the corresponding CHC ability.

	**(a) Dependent: VCI**				**(b) Dependent: WMI**			
**Model**	**Predictors**	**B**	**5% LLCI**	**95% ULCI**	**Predictors**	**B**	**5% LLCI**	**95% ULCI**
1a	Gc (M)	0.06	−0.14	0.27	Gsm (M)	0.16	−0.04	0.35
	Gc (F)	0.13	−0.06	0.30	Gsm (F)	0.24	0.03	0.43
1b	Gc (M)	−0.08	−0.29	0.14	Gsm (M)	0.19	−0.02	0.38
	Gc (F)	0.04	−0.19	0.26	Gsm (F)	0.19	−0.05	0.43
	Edu (M)	0.38	0.18	0.57	Edu (M)	−0.07	−0.30	0.18
	Edu (F)	0.01	−0.26	0.28	Edu (F)	0.11	−0.12	0.33
2	Gc (M)	−0.06	−0.28	0.20	Gsm (M)	0.18	−0.16	0.46
	Gc (F)	0.08	−0.17	0.34	Gsm (F)	0.38	0.08	0.62
	Sex (C)	0.26	−0.35	0.85	Sex (C)	−0.06	−0.40	0.37
	Edu (M)	0.05	−0.25	0.28	Edu (M)	0.02	−0.33	0.30
	Edu (F)	0.21	−0.10	0.51	Edu (F)	0.06	−0.21	0.35
	Sex (C) × Edu (M)	0.69	0.12	1.30	Sex (C) × Edu (M)	−0.14	−0.58	0.32
	Sex (C) × Edu (F)	−0.47	−1.23	0.22	Sex (C) × Edu (F)	−0.16	−0.63	0.34
	Sex (C) × Gc (M)	−0.14	−0.77	0.28	Sex (C) × Gsm (M)	−0.74	−1.18	−0.19
	Sex (C) × Gc (F)	−0.12	−0.71	0.47	Sex (C) × Gsm (F)	0.22	−0.35	0.73
	**(c) Dependent: PRI ^‡^**				**(d) Dependent: PSI**			
**Model**	**Predictors**	**B**	**5% LLCI**	**95% ULCI**	**Predictors**	**B**	**5% LLCI**	**95% ULCI**
1a	Gf (M) [Gv (M)]	0.20 (0.17)	0.02 (−0.02)	0.39 (0.35)	Gs (M)	0.30	0.11	0.48
	Gf (F) [Gv (F)]	−0.12 (0.20)	−0.34 (0.02)	0.09 (0.35)	Gs (F)	−0.11	−0.30	0.09
1b	Gf (M) [Gv (M)]	0.12 (0.12)	−0.07 (−0.09)	0.34 (0.32)	Gs (M)	0.30	0.11	0.49
	Gf (F) [Gv (F)]	−0.21 (−0.21)	−0.39 (−0.39)	−0.01 (−0.01)	Gs (F)	−0.09	−0.29	0.11
	Edu (M)	0.18 (0.18)	−0.05 (−0.05)	0.39 (0.39)	Edu (M)	0.08	−0.11	0.28
	Edu (F)	0.15 (0.15)	−0.14 (−0.14)	0.43 (0.43)	Edu (F)	−0.15	−0.36	0.05
2	Gf (M) [Gv (M)]	0.22 (0.22)	−0.05 (−0.05)	0.57 (0.57)	Gs (M)	0.28	0.06	0.47
	Gf (F) [Gv (F)]	−0.36 (−0.36)	−0.59 (−0.59)	−0.17 (−0.17)	Gs (F)	−0.21	−0.44	0.02
	Sex (C)	−0.48 (−0.48)	−1.09 (−1.09)	0.11 (0.11)	Sex (C)	−0.44	−1.03	0.05
	Edu (M)	0.21 (0.21)	−0.08 (−0.08)	0.45 (0.45)	Edu (M)	0.33	0.10	0.57
	Edu (F)	0.10 (0.10)	−0.29 (−0.29)	0.48 (0.48)	Edu (F)	−0.29	−0.52	−0.06
	Sex (C) × Edu (M)	−0.31 (−0.31)	−0.84 (−0.84)	0.44 (0.44)	Sex (C)xEdu (M)	−0.40	−0.83	0.09
	Sex (C) × Edu (F)	0.21 (0.21)	−0.49 (−0.49)	0.81 (0.81)	Sex (C)xEdu (F)	−0.14	−0.78	0.47
	Sex (C) × Gf (M)	0.53 (0.53)	−0.15 (−0.15)	1.42 (1.42)	Sex (C)xGs (M)	0.47	−0.11	1.03
	Sex (C) × Gf (F)	−0.21 (−0.21)	−1.14 (−1.14)	0.60 (0.60)	Sex (C)xGs (F)	0.34	−0.24	1.00

Legend: VCI = Verbal Comprehension Index; PRI = Perceptual Reasoning Index; WMI = Working Memory Index; PSI = Processing Speed Index; Gs = Processing Speed; Gc = Comprehension-Knowledge; Gf = Fluid Reasoning; Gv = Visual Processing; Gsm = Short-Term Memory; (C) = children variables; (M) = mother variables; (F) = father variables; Edu = Education; B = regression coefficients; 5% LLCI = lower limit confidence interval; 95% ULCI = upper limit confidence interval. Note: ^‡^ = For PRI analyses the values in parentheses indicate that the predictor is Gv, while those outside the parentheses indicate that the predictor is Gf.

**Table 5:** PLS path models. Model fit for Models 3 and 4. The dependent variables are children’s WISC-IV scores, and the predictors include parental cognitive abilities, personality traits, education, children’s biological sex, and its interactions with parental variables that emerged as the best predictors in Models 1 and 2.

**(a) Dependent: VCI**					**(b) Dependent: WMI**				
Model	R^2^	SRMR	Q^2^	BIC	Model	R^2^	SRMR	Q^2^	BIC
3a	0.21	0.080	0.06	4.38	3a	0.11	0.080	0.00	12.49
3b	0.14	0.078	0.12	−2.27	3b	0.04	0.078	0.02	4.66
4	0.21	0.074	0.06	4.38	4	0.11	0.074	0.00	12.49
**(c) Dependent: PRI**					**(d) Dependent: PSI**				
Model	R^2^	SRMR	Q^2^	BIC	Model	R^2^	SRMR	Q^2^	BIC
3a	0.07	0.080	0.01	6.94	3a	0.10	0.080	0.02	9.01
3b	0.19	0.078	0.13	1.93	3b	---	---	---	---
4	0.21	0.074	0.10	8.53	4	0.10	0.074	0.02	9.01

Legend: VCI = Verbal Comprehension Index; PRI = Perceptual Reasoning Index; WMI = Working Memory Index; PSI = Processing Speed Index.

## Data Availability

The data presented in this study are openly available in OSF at https://osf.io/th3sq (accessed on 1 December 2024).

## Supplementary Materials

The following supporting information can be downloaded at: https://www.mdpi.com/article/10.3390/jintelligence13020012/s1. Table S1. Correlation matrix of children’s WISC-IV Scores with parents’ education and personality traits method: Pearson (parametric) and Spearman (non-parametric) methods Content: Correlation data between children’s cognitive scores and parental education/personality traits. Table S2. PLS regressions—model fit for basic and moderation analyses content: Dependent variables are children’s WISC-IV scores; predictors include parental personality traits, education, child’s biological sex, and interactions. Metrics: R^2^, SRMR, Q^2^, BIC for each model. Table S3. Bootstrap tests of model coefficients (parental extroversion and education effects) content: Dependent variables include children’s WISC-IV scores, examining predictors such as parental extroversion and education. Table S4. Bootstrap tests of model coefficients (parental agreeableness and education effects) content: Includes coefficients and confidence intervals for effects of parental agreeableness on children’s cognitive abilities. Table S5. Bootstrap tests of model coefficients (parental conscientiousness and education effects) content: Examines relationships between parental conscientiousness and children’s WISC-IV scores. Table S6. Bootstrap tests of model coefficients (parental neuroticism and education effects) content: Analyzes the impact of parental neuroticism on children’s cognitive scores. Table S7. Bootstrap tests of model coefficients (parental openness and education effects) content: Investigates effects of parental openness on children’s WISC-IV scores. Table S8. Bootstrap tests for model 3a content: Explores relationships between selected parental cognitive abilities, education, and child-specific factors. Table S9. Bootstrap tests for model 3b content: Focus on parental personality traits, education, and their interactions with children’s WISC-IV scores. Table S10. Bootstrap tests for model 4 content: Comprehensive model including parental cognitive abilities, personality traits, education, and their interactions with child outcomes. Supplementary Figure S1. Path diagrams for models 3a and 3b. Description: Panel a: Prediction of children’s WISC-IV scores based on mothers’ and fathers’ CHC abilities and education. Panel b: Prediction of WISC-IV scores using parental personality traits and educational levels. Metrics: Standardized regression coefficients with bootstrap significance levels.
