# Supplementary material for: The Role of Parental Education, Intelligence, and Personality on the Cognitive Abilities of Gifted Children
Source: J Intell. 2025 Jan 21;13(2):12. doi: 10.3390/jintelligence13020012 (PMC11856753; doi:10.3390/jintelligence13020012)
Supplement: Supplementary file 1 [file jintelligence-13-00012-s001.zip › jintelligence-3263916-supplementary.pdf]

Supplementary Materials

The Role of Parental Education, Intelligence, and Personality on the Cognitive Abilities of Gifted Children

Lina Pezzuti [1], Morena Farese [1], James Dawe [2] and Marco Lauriola [2,*]

[1] Department of Dynamic and Clinical Psychology and Health Studies, Sapienza University of Rome, 00185 Rome, Italy.
[2] Department of Social and Developmental Psychology, Sapienza University of Rome, 00185 Rome, Italy
* Correspondence: marco.lauriola@uniroma1.it

List of Supplementary Tables and figures

S1	Correlation Matrix of Children's WISC-IV Scores with Parents' Education and Personality Traits
S2	PLS Regressions – Model Fit for Basic and Moderation Analyses
S3	Bootstrap Tests of Model Coefficients (Parental Extroversion and Education Effects)
S4	Bootstrap Tests of Model Coefficients (Parental Agreeableness and Education Effects)
S5	Bootstrap Tests of Model Coefficients (Parental Conscientiousness and Education Effects)
S6	Bootstrap Tests of Model Coefficients (Parental Neuroticism and Education Effects)
S7	Bootstrap Tests of Model Coefficients (Parental Openness and Education Effects)
S8	Bootstrap Tests for Model 3a
S9	Bootstrap Tests for Model 3b
S10	Bootstrap Tests for Model 4
F1	Path diagram for models 3a and 3b.

Supplementary Table S1. Correlation Matrix of Children WISC-IV scores with Mother's and Father's Education and Personality Traits using Pearson (parametric) and Spearman (non-parametric) methods**.**

	1.	2.	3.	4.	5.	6.	7.	8.	9.	10.	11.	12.	13.	14.	15.	16.
**1. VCI (C)**	--	.17	-.12	-.12	.36**	.13	.13	.00	-.14	-.04	.09	-.09	.01	-.14	-.07	-.14
**2. PRI(C)**	.18	--	.33**	.18	.17	.02	-.03	.39**	-.11	.05	.11	.17	.29**	-.11	-.14	.19
**3. WMI(C)**	-.12	.32**	--	.22*	-.03	.08	-.07	.19	.11	.04	.13	-.07	.19	-.13	-.02	.06
**4. PSI(C)**	-.13	.17	.26*	--	-.02	.11	.09	.10	.13	-.06	-.17	-.07	.10	.12	-.01	.07
**5. Edu (M)**	.37**	.23*	-.02	.01	--	.25*	-.07	-.01	-.15	.23*	.24*	.17	-.01	-.12	-.27*	-.11
**6. E (M)**	.09	.06	.09	.15	.25*	--	.28*	.16	-.24*	-.12	.23*	.05	-.02	.00	-.17	.16
**7. A (M)**	.20	.00	-.02	.10	-.03	.25*	--	-.04	-.25*	-.33**	-.11	-.07	-.22*	.21*	-.35**	.11
**8. C (M)**	.01	.36**	.16	.10	.05	.23*	-.01	--	-.19	.11	.09	.03	.10	-.24*	.15	.26*
**9. N (M)**	-.15	-.13	.15	.10	-.21*	-.12	-.22*	-.19	--	-.02	-.06	-.34**	.20	-.07	-.04	.07
**10. O (M)**	.01	.12	.01	-.07	.25*	-.10	-.28*	.16	-.06	--	-.04	-.07	-.13	-.46**	.38**	-.13
**11. Edu (F)**	.09	.12	.14	-.18	.26*	.18	-.06	.11	-.04	-.05	--	.01	.12	.05	-.26*	.23*
**12. E (F)**	-.09	.20	-.06	-.10	.22*	.09	-.09	.01	-.31**	-.08	.03	--	.25*	.17	-.17	.37**
**13. A (F)**	-.03	.29*	.20	.13	.01	.04	-.15	.08	.19	-.20	.05	.21*	--	.14	-.29**	.20
**14. C (F)**	-.14	-.15	-.15	.02	-.15	-.08	.17	-.34**	-.06	-.43**	-.04	.11	.11	--	-.25*	.16
**15. N (F)**	-.08	-.11	-.02	.04	-.32**	-.16	-.31**	.18	-.12	.32**	-.25*	-.19	-.34**	-.27*	--	-.17
**16. O (F)**	-.15	.17	.07	.02	-.06	.18	.10	.21*	.07	-.19	.26*	.39**	.19	.08	-.20	--

** Correlation is significant at the 0.01 level (one-tailed).
* Correlation is significant at the 0.05 level (one-tailed).
Note: Pearson and Spearman correlations are below and above the diagonal, respectively
Legend: VCI = Verbal Comprehension Index; PRI = Perceptual Reasoning Index; WMI = Working Memory Index; PSI = Processing Speed Index; E = Extroversion; A = Agreeableness; C = Conscientiousness; N = Neuroticism; O = Openess; (C) = Children variables; (M) = Mother variables; (F) = Father variables

Supplementary Table S2. PLS Regressions. Model fit for basic and moderation analyses. Dependent variables are children WISC-IV scores, predictors are parental Personality traits and Education, children sex and its interactions with parental personality traits.

**Predictor: Extroversion (E)**																			
**Dependent: VCI**					**Dependent: WMI**					**Dependent: PRI**					**Dependent: PSI**				
Model	R^2	SRM R	Q^2	BIC	Model	R^2	SRM R	Q^2	BIC	Model	R^2	SRM R	Q^2	BIC	Model	R^2	SRM R	Q^2	BIC
1a	.01	.00†	-.05	10757	1a	.01	.00†	-.06	10739	1a	.02	.00†	-.05	10213	1a	.04	.00†	-.02	8890
1b	.16	.00†	.05	8764	1b	.03	.00†	-.10	18032	1b	.06	.00†	-.10	15571	1b	.07	.00†	-.05	15418
2	.27	.08	-.10	19578	2	.03	.01	-.30	38738	2	.12	.05	-.31	32273	2	.23	.09	-.12	23774
**Predictor: Agreeableness (A)**																			
**Dependent: VCI**					**Dependent: WMI**					**Dependent: PRI**					**Dependent: PSI**				
Model	R^2	SRM R	Q^2	BIC	Model	R^2	SRM R	Q^2	BIC	Model	R^2	SRM R	Q^2	BIC	Model	R^2	SRM R	Q^2	BIC
1a	.03	.00†	-.03	9384	1a	.04	.00†	.00	8843	1b	.04	.00†	-.01	8572	1a	.03	.00†	-.03	9735
1b	.17	.00†	.07	7320	1b	.06	.00†	-.05	16221	1d	.10	.00†	-.04	13172	1b	.05	.00†	-.07	16592
2	.30	.08	-.07	17055	2	.07	.02	-.22	36285	2	.17	.06	-.17	28587	2	.17	.09	-.17	28449
**Predictor: Conscientiousness (C)**																			
**Dependent: VCI**					**Dependent: WMI**					**Dependent: PRI**					**Dependent: PSI**				
Model	R^2	SRM R	Q^2	BIC	Model	R^2	SRM R	Q^2	BIC	Model	R^2	SRM R	Q^2	BIC	Model	R^2	SRM R	Q^2	BIC
1a	.01	.00†	-.08	10938	1a	.03	.00†	-.03	9676	1a	.11	.00†	.06	4016	1a	.02	.00†	-.09	10533
1b	.14	.00†	.01	10163	1b	.05	.00†	-.08	16823	1b	.15	.00†	.04	9010	1b	.04	.00†	-.12	17351
2	.29	.08	-.12	18187	2	.06	.02	-.27	36650	2	.30	.07	.03	17230	2	.23	.10	-.15	23450

(continues)

Predictor: Neuroticism (N)																			
**Dependent: VCI**					**Dependent: WMI**					**Dependent: PRI**					**Dependent: PSI**				
Model	R^2	SRMR	Q^2	BIC	Model	R^2	SRMR	Q^2	BIC	Model	R^2	SRMR	Q^2	BIC	Model	R^2	SRMR	Q^2	BIC
1a	.05	.00†	-.01	8364	1a	.03	.00†	-.05	9786	1a	.04	.00†	-.02	9094	1a	.01	.00†	-.07	10995
1b	.15	.00†	.03	9542	1b	.04	.00†	-.09	17154	1b	.07	.00†	-.07	15267	1b	.03	.00†	-.12	17894
2	.30	.08	-.12	17021	2	.06	.02	-.26	37179	2	.17	.08	-.33	28897	2	.17	.09	-.27	28756
**Predictor: Openness (O)**																			
**Dependent: VCI**					**Dependent: WMI**					**Dependent: PRI**					**Dependent: PSI**				
Model	R^2	SRMR	Q^2	BIC	Model	R^2	SRMR	Q^2	BIC	Model	R^2	SRMR	Q^2	BIC	Model	R^2	SRMR	Q^2	BIC
1a	.01	.00†	-.06	10859	1a	.00	.00†	-.04	11351	1a	.04	.00†	-.05	9007	1a	.00	.00†	-.09	11452
1b	.14	.00†	.02	9898	1b	.02	.00†	-.09	18685	1b	.09	.00†	-.07	13915	1b	.03	.00†	-.11	19021
2	.28	.09	-.08	19549	2	.04	.02	-.19	37891	2	.14	.05	-.25	30677	2	.15	.09	-.33	30407

Legend: VCI = Verbal Comprehension Index; PRI = Perceptual Reasoning Index; WMI = Working Memory Index; PSI = Processing Speed Index;

Note: † = Saturated model has SRMR equal to 0.00.

Supplementary Table S3. PLS Regressions. Bootstrap tests of model coefficients for basic and moderation analyses. Dependent variables are children WISC-IV scores, predictors are parental Extroversion and Education, children sex and its interactions with parental personality traits.

	**Dependent: VCI**					**Dependent: WMI**			
**Model**	**Predictors**	**B**	**5% LLCI**	**95% ULCI**	**Model**	**Predictors**	**B**	**5% LLCI**	**95% ULCI**
**1a**	**E (M)**	.10	-.10	.29	**1a**	**E (M)**	.09	-.14	.30
	**E (F)**	-.06	-.26	.15		**E (F)**	-.07	-.28	.13
**1b**	**E (M)**	.00	-.19	.17	**1b**	**E (M)**	.08	-.15	.28
	**E (F)**	-.14	-.34	.07		**E (F)**	-.07	-.28	.14
	**Edu (M)**	.39	.20	.57		**Edu (M)**	-.07	-.29	.17
	**Edu (F)**	.03	-.18	.24		**Edu (F)**	.13	-.06	.32
**2**	**E (M)**	-.08	-.29	.13	**2**	**E (M)**	.09	-.26	.38
	**E (F)**	-.06	-.31	.17		**E (F)**	-.05	-.37	.25
	**Sex (C)**	.36	-.18	1.06		**Sex (C)**	-.07	-.41	.34
	**Edu (M)**	.08	-.21	.33		**Edu (M)**	-.04	-.41	.30
	**Edu (F)**	.24	-.03	.52		**Edu (F)**	.14	-.14	.41
	**Sex (C) × Edu (F)**	.53	-.09	1.02		**Sex (C) × Edu (F)**	-.06	-.57	.63
	**Sex (C) × Edu (M)**	-.48	-1.23	.02		**Sex (C) × Edu (M)**	-.03	-.53	.48
	**Sex (C) × E (M)**	-.04	-.67	.48		**Sex (C) × E (M)**	-.06	-.64	.47
	**Sex (C) × E (F)**	.15	-.24	.62		**Sex (C) × E (F)**	-.03	-.48	.38
	**Dependent: PRI**					**Dependent: PSI**			
**Model**	**Predictors**	**B**	**5% LLCI**	**95% ULCI**	**Model**	**Predictors**	**B**	**5% LLCI**	**95% ULCI**
**1a**	**E (M)**	.03	-.15	.21	**1a**	**E (M)**	.16	-.04	.34
	**E (F)**	.14	-.07	.33		**E (F)**	-.13	-.31	.05
**1b**	**E (M)**	-.03	-.23	.17	**1b**	**E (M)**	.18	-.03	.38
	**E (F)**	.09	-.13	.29		**E (F)**	-.13	-.33	.05
	**Edu (M)**	.19	-.02	.40		**Edu (M)**	.05	-.15	.27

	**Edu (F)**	.07	-.18	.34		**Edu (F)**	-.17	-.40	.05
**2**	**E (M)**	-.06	-.33	.18	**2**	**E (M)**	.14	-.15	.37
	**E (F)**	.09	-.18	.34		**E (F)**	-.11	-.32	.11
	**Sex (C)**	-.56	-1.18	-.02		**Sex (C)**	-.41	-1.13	.19
	**Edu (M)**	.20	-.12	.46		**Edu (M)**	.33	.09	.58
	**Edu (F)**	.07	-.24	.45		**Edu (F)**	-.39	-.62	-.14
	**Sex (C) × Edu (F)**	.19	-.31	.73		**Sex (C) × Edu (F)**	-.40	-.85	.12
	**Sex (C) × Edu (M)**	-.18	-1.02	.33		**Sex (C) × Edu (M)**	.10	-.38	.61
	**Sex (C) × E (M)**	.23	-.35	.96		**Sex (C) × E (M)**	-.35	-.92	.37
	**Sex (C) × E (F)**	.19	-.31	.75		**Sex (C) × E (F)**	.76	.29	1.31

Legend: E = Extroversion; VCI = Verbal Comprehension Index; PRI = Perceptual Reasoning Index; WMI = Working Memory Index; PSI = Processing Speed Index; Gs = processing speed; Gc = Comprehension-Knowledge; Gf = Fluid reasoning; Gv = Visual processing; Gsm = Short-Term memory; (C) = Children variables; (M) = Mother variables; (F) = Father variables; B = regression coefficients; 5% LLCI = Lower Limit Confidence Interval; 95% ULCI = Upper Limit Confidence Interval.

Supplementary Table S4. PLS Regressions. Bootstrap tests of model coefficients for basic and moderation analyses. Dependent variables are children WISC-IV scores, predictors are parental Agreeableness and Education, children sex and its interactions with parental personality traits.

	**Dependent: VCI**					**Dependent: WMI**			
**Model**	**Predictors**	**B**	**5% LLCI**	**95% ULCI**	**Model**	**Predictors**	**B**	**5% LLCI**	**95% ULCI**
**1a**	**A (M)**	.17	-.02	.35	**1a**	**A (M)**	.01	-.19	.20
	**A (F)**	-.03	-.22	.17		**A (F)**	.20	.03	.36
**1b**	**A (M)**	.19	.01	.35	**1b**	**A (M)**	.02	-.19	.21
	**A (F)**	-.03	-.22	.15		**A (F)**	.19	.00	.36
	**Edu (M)**	.36	.18	.52		**Edu (M)**	-.06	-.29	.18
	**Edu (F)**	.04	-.17	.25		**Edu (F)**	.13	-.06	.32
**2**	**A (M)**	.20	.03	.37	**2**	**A (M)**	.03	-.27	.30
	**A (F)**	.09	-.16	.33		**A (F)**	.25	-.03	.49
	**Sex (C)**	.27	-.28	.90		**Sex (C)**	-.01	-.42	.46
	**Edu (M)**	.06	-.20	.28		**Edu (M)**	-.05	-.44	.28
	**Edu (F)**	.25	-.02	.50		**Edu (F)**	.17	-.08	.42
	**Sex × Edu (F)**	-.22	-.80	.20		**Sex × Edu (F)**	-.16	-.60	.45
	**Sex (C) × Edu (M)**	.58	.07	1.06		**Sex (C) × Edu (M)**	-.05	-.63	.52
	**Sex (C) × A (M)**	-.28	-.78	.26		**Sex (C) × A (M)**	-.08	-.64	.39
	**Sex (C) × A (F)**	-.46	-1.10	.09		**Sex (C) × A (F)**	-.15	-.55	.24
	**Dependent: PRI**					**Dependent: PSI**			
**Model**	**Predictors**	**B**	**5% LLCI**	**95% ULCI**	**Model**	**Predictors**	**B**	**5% LLCI**	**95% ULCI**
**1a**	**A (M)**	.03	-.15	.21	**1a**	**A (M)**	.11	-.07	.28
	**A (F)**	.21	.01	.41		**A (F)**	.14	-.07	.34
**1b**	**A (M)**	.04	-.15	.22	**1b**	**A (M)**	.10	-.08	.27
	**A (F)**	.21	-.02	.40		**A (F)**	.15	-.06	.35
	**Edu (M)**	.21	.01	.40		**Edu (M)**	.06	-.13	.26

	**Edu (F)**	.05	-.19	.31		**Edu (F)**	-.16	-.36	.05
**2**	**A (M)**	.20	-.03	.42	**2**	**A (M)**	.12	-.12	.33
	**A (F)**	.20	-.06	.42		**A (F)**	.03	-.20	.28
	**Sex (C)**	-.39	-.90	.18		**Sex (C)**	-.51	-1.09	-.01
	**Edu (M)**	.23	-.07	.48		**Edu (M)**	.33	.10	.56
	**Edu (F)**	.06	-.24	.43		**Edu (F)**	-.34	-.55	-.11
	**Sex × Edu (F)**	.01	-.70	.62		**Sex × Edu (F)**	-.40	-.87	.15
	**Sex (C) × Edu (M)**	-.46	-1.09	-.01		**Sex (C) × Edu (M)**	.17	-.25	.74
	**Sex (C) × A (M)**	-.21	-.81	.36		**Sex (C) × A (M)**	.39	-.25	1.00
	**Sex (C) × A (F)**	.17	-.27	.71		**Sex (C) × A (F)**	.23	-.31	.68

Legend: A = Agreeableness; VCI = Verbal Comprehension Index; PRI = Perceptual Reasoning Index; WMI = Working Memory Index; PSI = Processing Speed Index; Gs = processing speed; Gc = Comprehension-Knowledge; Gf = Fluid reasoning; Gv = Visual processing; Gsm = Short-Term memory; (C) = Children variables; (M) = Mother variables; (F) = Father variables; B = regression coefficients; 5% LLCI = Lower Limit Confidence Interval; 95% ULCI = Upper Limit Confidence Interval.

Supplementary Table S5. PLS Regressions. Bootstrap tests of model coefficients for basic and moderation analyses. Dependent variables are children WISC-R scores, predictors are parental Conscientiousness and Education, children sex and its interactions with parental personality traits.

	**Dependent: VCI**					**Dependent: WMI**			
**Model**	**Predictors**	**B**	**5% LLCI**	**95% ULCI**	**Model**	**Predictors**	**B**	**5% LLCI**	**95% ULCI**
**1a**	**C (M)**	-.05	-.28	.19	**1a**	**C (M)**	.14	-.08	.34
	**C (F)**	-.11	-.34	.14		**C (F)**	-.06	-.27	.16
**1b**	**C (M)**	-.04	-.25	.19	**1b**	**C (M)**	.13	-.10	.34
	**C (F)**	-.02	-.24	.21		**C (F)**	-.07	-.29	.15
	**Edu (M)**	.36	.17	.52		**Edu (M)**	-.09	-.32	.15
	**Edu (F)**	.03	-.18	.24		**Edu (F)**	.14	-.06	.32
**2**	**C (M)**	.02	-.24	.29	**2**	**C (M)**	.21	-.18	.59
	**C (F)**	.08	-.17	.32		**C (F)**	-.04	-.34	.29
	**Sex (C)**	.32	-.37	.86		**Sex (C)**	-.14	-.57	.39
	**Edu (M)**	.06	-.20	.29		**Edu (M)**	-.12	-.54	.25
	**Edu (F)**	.23	-.01	.50		**Edu (F)**	.17	-.07	.44
	**Sex (C) × Edu (F)**	-.38	-.88	.21		**Sex (C) × Edu (F)**	.05	-.69	.82
	**Sex (C) × Edu (M)**	-.58	-1.16	-.17		**Sex (C) × Edu (M)**	-.18	-.67	.21
	**Sex (C) × C (M)**	.52	.02	1.58		**Sex (C) × C (M)**	-.15	-.62	.42
	**Sex (C) × C (F)**	-.10	-.64	.86		**Sex (C) × C (F)**	-.18	-.73	.52
	**Dependent: PRI**					**Dependent: PSI**			
**Model**	**Predictors**	**B**	**5% LLCI**	**95% ULCI**	**Model**	**Predictors**	**B**	**5% LLCI**	**95% ULCI**
**1a**	**C (M)**	.32	.12	.52	**1a**	**C (M)**	.13	-.06	.32
	**C (F)**	-.01	-.21	.21		**C (F)**	.09	-.20	.39
**1b**	**C (M)**	.33	.13	.52	**1b**	**C (M)**	.14	-.05	.33
	**C (F)**	.04	-.16	.24		**C (F)**	.10	-.21	.42
	**Edu (M)**	.20	.02	.37		**Edu (M)**	.07	-.13	.29

	**Edu (F)**	.05	-.19	.30		**Edu (F)**	-.16	-.37	.05
**2**	**C (M)**	.56	.31	.79	**2**	**C (M)**	.14	-.13	.39
	**C (F)**	.18	-.04	.40		**C (F)**	-.08	-.40	.33
	**Sex (C)**	-.72	-1.33	-.17		**Sex (C)**	-.60	-1.24	.00
	**Edu (M)**	.10	-.17	.32		**Edu (M)**	.25	-.05	.53
	**Edu (F)**	.05	-.23	.39		**Edu (F)**	-.33	-.56	-.08
	**Sex (C) × Edu (F)**	-.39	-.88	.05		**Sex (C) × Edu (F)**	.23	-.25	.94
	**Sex (C) × Edu (M)**	.49	.08	1.15		**Sex (C) × Edu (M)**	.66	.12	1.23
	**Sex (C) × C (M)**	-.10	-.69	.38		**Sex (C) × C (M)**	.58	.07	1.09
	**Sex (C) × C (F)**	-.25	-.70	.26		**Sex (C) × C (F)**	-.07	-.69	.71

Legend: C = Conscientiousness; VCI = Verbal Comprehension Index; PRI = Perceptual Reasoning Index; WMI = Working Memory Index; PSI = Processing Speed Index; Gs = processing speed; Gc = Comprehension-Knowledge; Gf = Fluid reasoning; Gv = Visual processing; Gsm = Short-Term memory; (C) = Children variables; (M) = Mother variables; (F) = Father variables; B = regression coefficients; 5% LLCI = Lower Limit Confidence Interval; 95% ULCI = Upper Limit Confidence Interval.

Supplementary Table S6. PLS Regressions. Bootstrap tests of model coefficients for basic and moderation analyses. Dependent variables are children WISC-IV scores, predictors are parental Neuroticism and Education, children sex and its interactions with parental personality traits.

	**Dependent: VCI**					**Dependent: WMI**			
**Model**	**Predictors**	**B**	**5% LLCI**	**95% ULCI**	**Model**	**Predictors**	**B**	**5% LLCI**	**95% ULCI**
**1a**	**N (M)**	-.18	-.35	.00	**1a**	**N (M)**	.14	-.09	.35
	**N (F)**	-.15	-.33	.03		**N (F)**	-.06	-.26	.15
**1b**	**N (M)**	-.10	-.28	.09	**1b**	**N (M)**	.14	-.09	.36
	**N (F)**	-.05	-.25	.16		**N (F)**	-.04	-.26	.18
	**Edu (M)**	.33	.13	.52		**Edu (M)**	-.04	-.30	.20
	**Edu (F)**	.02	-.20	.23		**Edu (F)**	.13	-.06	.32
**2**	**N (M)**	-.17	-.33	.02	**2**	**N (M)**	.13	-.16	.44
	**N (F)**	-.17	-.36	.04		**N (F)**	-.12	-.40	.20
	**Sex (C)**	.34	-.27	1.13		**Sex (C)**	-.07	-.47	.43
	**Edu (M)**	-.02	-.26	.21		**Edu (M)**	-.04	-.48	.29
	**Edu (F)**	.24	-.04	.50		**Edu (F)**	.14	-.10	.43
	**Sex (C) × Edu (F)**	.39	-.39	.91		**Sex (C) × Edu (F)**	.28	-.29	.78
	**Sex (C) × Edu (M)**	.32	-.37	1.01		**Sex (C) × Edu (M)**	-.01	-.54	.54
	**Sex (C) × N (M)**	-.42	-1.24	.17		**Sex (C) × N (M)**	.06	-.61	.73
	**Sex (C) × N (F)**	.75	.14	1.31		**Sex (C) × N (F)**	-.01	-.62	.49
	**Dependent: PRI**					**Dependent: PSI**			
**Model**	**Predictors**	**B**	**5% LLCI**	**95% ULCI**	**Model**	**Predictors**	**B**	**5% LLCI**	**95% ULCI**
**1a**	**N (M)**	-.15	-.35	.06	**1a**	**N (M)**	.09	-.13	.31
	**N (F)**	-.14	-.34	.06		**N (F)**	.02	-.23	.24
**1b**	**N (M)**	-.11	-.31	.11	**1b**	**N (M)**	.10	-.14	.32
	**N (F)**	-.08	-.28	.14		**N (F)**	.00	-.29	.25
	**Edu (M)**	.17	-.03	.36		**Edu (M)**	.08	-.16	.30

	**Edu (F)**	.06	-.20	.34		**Edu (F)**	-.15	-.38	.08
**2**	**N (M)**	-.25	-.47	-.02	**2**	**N (M)**	.06	-.19	.28
	**N (F)**	-.13	-.36	.11		**N (F)**	.14	-.22	.44
	**Sex (C)**	-.53	-1.08	.08		**Sex (C)**	-.28	-1.25	.53
	**Edu (M)**	.15	-.14	.39		**Edu (M)**	.35	.05	.62
	**Edu (F)**	.05	-.27	.44		**Edu (F)**	-.34	-.55	-.09
	**Sex (C) × Edu (F)**	.44	-.21	1.33		**Sex (C) × Edu (F)**	-.50	-1.04	.19
	**Sex (C) × Edu (M)**	.18	-.50	1.07		**Sex (C) × Edu (M)**	-.39	-1.04	.24
	**Sex (C) × N (M)**	.45	-.07	1.04		**Sex (C) × N (M)**	.22	-.62	1.09
	**Sex (C) × N (F)**	.49	-.09	1.49		**Sex (C) × N (F)**	.44	-.12	1.06

Legend: N = Neuroticism; VCI = Verbal Comprehension Index; PRI = Perceptual Reasoning Index; WMI = Working Memory Index; PSI = Processing Speed Index; Gs = processing speed; Gc = Comprehension-Knowledge; Gf = Fluid reasoning; Gv = Visual processing; Gsm = Short-Term memory; (C) = Children variables; (M) = Mother variables; (F) = Father variables; B = regression coefficients; 5% LLCI = Lower Limit Confidence Interval; 95% ULCI = Upper Limit Confidence Interval.

Supplementary Table S7. PLS Regressions. Bootstrap tests of model coefficients for basic and moderation analyses. Dependent variables are children WISC-IV scores, predictors are parental Openness and Education, children sex and its interactions with parental personality traits.

	**Dependent: VCI**					**Dependent: WMI**			
**Model**	**Predictors**	**B**	**5% LLCI**	**95% ULCI**	**Model**	**Predictors**	**B**	**5% LLCI**	**95% ULCI**
**1a**	**O (M)**	.06	-.13	.26	**1a**	**O (M)**	-.01	-.22	.19
	**O (F)**	-.07	-.30	.17		**O (F)**	.05	-.12	.22
**1b**	**O (M)**	-.03	-.22	.18	**1b**	**O (M)**	.01	-.20	.22
	**O (F)**	-.07	-.30	.17		**O (F)**	.02	-.17	.20
	**Edu (M)**	.36	.17	.52		**Edu (M)**	-.06	-.31	.19
	**Edu (F)**	.04	-.17	.26		**Edu (F)**	.14	-.06	.33
**2**	**O (M)**	-.04	-.26	.17	**2**	**O (M)**	.05	-.22	.33
	**O (F)**	.01	-.30	.29		**O (F)**	.12	-.14	.37
	**Sex (C)**	.27	-.24	.88		**Sex (C)**	-.01	-.36	.42
	**Edu (M)**	.06	-.22	.28		**Edu (M)**	-.05	-.48	.27
	**Edu (F)**	.23	-.02	.49		**Edu (F)**	.15	-.10	.43
	**Sex (C) × Edu (F)**	-.03	-.57	.59		**Sex (C) × Edu (F)**	-.34	-.72	.03
	**Sex (C) × Edu (M)**	.53	-.02	1.05		**Sex (C) × Edu (M)**	-.12	-.69	.47
	**Sex (C) × O (M)**	-.51	-1.22	.07		**Sex (C) × O (M)**	.02	-.44	.53
	**Sex (C) × O (F)**	.31	-.25	.85		**Sex (C) × O (F)**	-.14	-.54	.31
	**Dependent: PRI**					**Dependent: PSI**			
**Model**	**Predictors**	**B**	**5% LLCI**	**95% ULCI**	**Model**	**Predictors**	**B**	**5% LLCI**	**95% ULCI**
**1a**	**O (M)**	.10	-.12	.32	**1a**	**O (M)**	-.03	-.24	.16
	**O (F)**	.18	-.02	.37		**O (F)**	.02	-.24	.27
**1b**	**O (M)**	.05	-.19	.26	**1b**	**O (M)**	-.06	-.27	.14
	**O (F)**	.19	-.07	.39		**O (F)**	.07	-.22	.32
	**Edu (M)**	.22	.01	.43		**Edu (M)**	.09	-.13	.30

	**Edu (F)**	.02	-.22	.31		**Edu (F)**	-.18	-.40	.07
**2**	**O (M)**	.11	-.16	.34	**2**	**O (M)**	-.08	-.31	.16
	**O (F)**	.21	-.10	.45		**O (F)**	.07	-.21	.34
	**Sex (C)**	-.53	-1.14	.20		**Sex (C)**	-.42	-1.12	.13
	**Edu (M)**	.17	-.18	.43		**Edu (M)**	.35	.09	.60
	**Edu (F)**	.04	-.25	.43		**Edu (F)**	-.38	-.60	-.13
	**Sex (C) × Edu (F)**	.34	-.21	.95		**Sex (C) × Edu (F)**	.61	.01	1.34
	**Sex (C) × Edu (M)**	.05	-.50	.78		**Sex (C) × Edu (M)**	-.07	-.71	.52
	**Sex (C) × O (M)**	-.06	-.80	.56		**Sex (C) × O (M)**	-.44	-.99	.26
	**Sex (C) × O (F)**	-.14	-.76	.55		**Sex (C) × O (F)**	-.17	-.96	.76

Legend: O = Openness; VCI = Verbal Comprehension Index; PRI = Perceptual Reasoning Index; WMI = Working Memory Index; PSI = Processing Speed Index; Gs = processing speed; Gc = Comprehension-Knowledge; Gf = Fluid reasoning; Gv = Visual processing; Gsm = Short-Term memory; (C) = Children variables; (M) = Mother variables; (F) = Father variables; B = regression coefficients; 5% LLCI = Lower Limit Confidence Interval; 95% ULCI = Upper Limit Confidence Interval.

Supplementary Figure 1. Path Diagrams. Panel a: Mothers' and fathers' CHC abilities and education were used to predict children's WISC-IV scores. Panel b: Mothers' and fathers' personality traits and educational levels were used to predict children's WISC-IV scores. Path coefficients are standardized regression coefficients. Bootstrap significance levels are indicated with asterisks: * p < .05; ** p < .01; *** p < .001 (one-tailed).

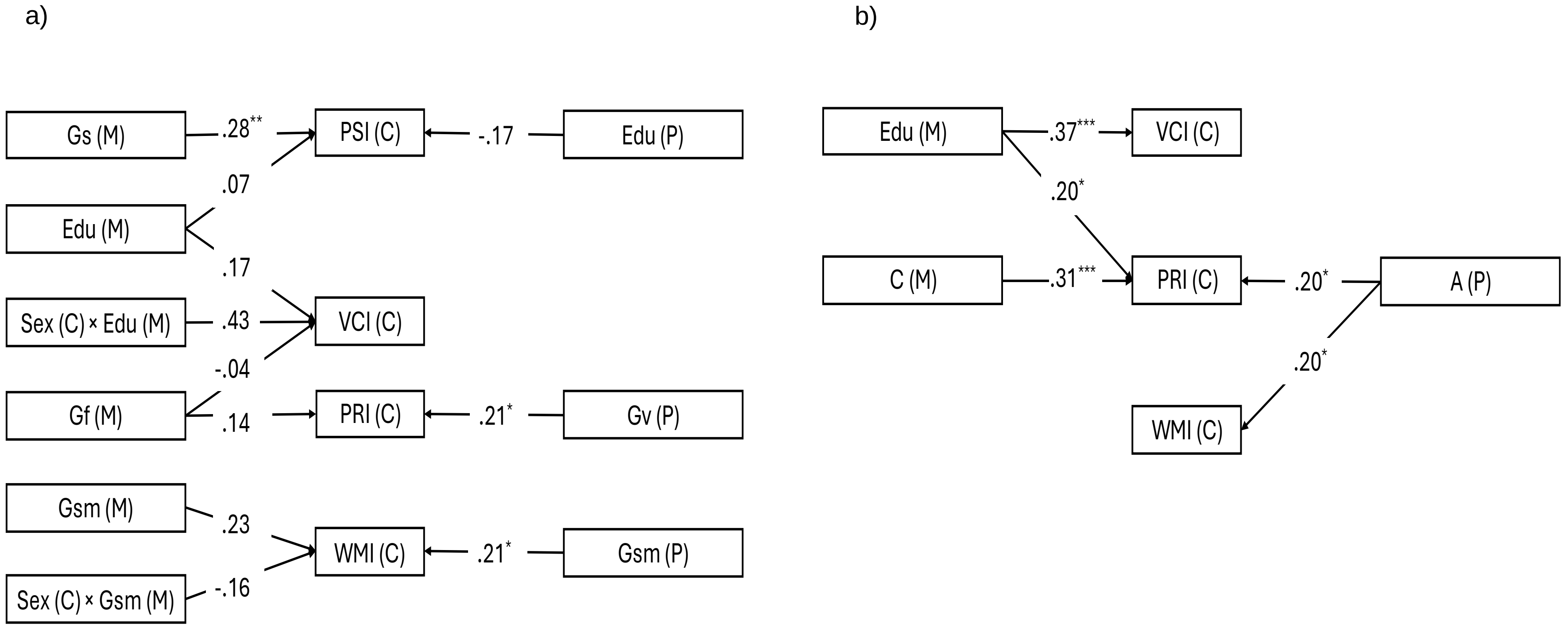

Supplementary Table S8. PLS Regressions. Bootstrap tests of model coefficients for Model 3a. Dependent variables are children WISC-IV scores, predictors are parental selected parental cognitive abilities, education, children sex and its interactions with parental personality abilities.

Path Coefficients	B	LLCI	ULCI
**Gf (M) → VCI (C)**	-.04	-.26	.23
**Gf (M) → PRI (C)**	.14	-.05	.34
**Gs (M) → PSI (C)**	.28	.10	.44
**Gsm (M) → WMI (C)**	.24	-.06	.51
**Gsm (P) → WMI (C)**	.21	.02	.42
**Gv (P) → PRI (C)**	.21	.02	.38
**Sex (C) → VCI (C)**	.33	-.14	.87
**Sex (C) → WMI (C)**	-.15	-.55	.23
**Edu (M) → VCI (C)**	.18	-.06	.38
**Edu (M) → PSI (C)**	.07	-.12	.26
**Edu (P) → PSI (C)**	-.17	-.38	.03
**Sex (C) × Edu (M) → VCI (C)**	.43	-.03	.80
**Sex (C) × Gsm (M) → WMI (C)**	-.16	-.53	.27

Legend: VCI = Verbal Comprehension Index; PRI = Perceptual Reasoning Index; WMI = Working Memory Index; PSI = Processing Speed Index; Gs = processing speed; Gc = Comprehension-Knowledge; Gf = Fluid reasoning; Gv = Visual processing; Gsm = Short-Term memory; (C) = Children variables; (M) = Mother variables; (F) = Father variables; B = regression coefficients; 5% LLCI = Lower Limit Confidence Interval; 95% ULCI = Upper Limit Confidence Interval.

Supplementary Table S9. PLS Regressions. Bootstrap tests of model coefficients for Model 3b. Dependent variables are children WISC-IV scores, predictors are parental selected personality traits, education, children sex and its interactions with parental personality abilities.

Path Coefficients	B	LLCI	ULCI
**A (P) - WMI (C)**	.20	.03	.36
**A (P) - PRI (C)**	.20	.01	.40
**C (M) - PRI (C)**	.31	.14	.48
**Edu (M) - VCI (C)**	.37	.21	.51
**Edu (M) - PRI (C)**	.20	.04	.38

Legend: VCI = Verbal Comprehension Index; PRI = Perceptual Reasoning Index; WMI = Working Memory Index; PSI = Processing Speed Index; A = Agreeableness; C = Conscientiousness; (C) = Children variables; (M) = Mother variables; (F) = Father variables; B = regression coefficients; 5% LLCI = Lower Limit Confidence Interval; 95% ULCI = Upper Limit Confidence Interval.

Supplementary Table S10. PLS Regressions. Bootstrap tests of model coefficients for Model 4. Dependent variables are children WISC-IV scores, predictors are parental selected parental cognitive abilities, personality traits, education, children sex and its interactions with parental personality abilities.

Path Coefficients	B	LLCI	ULCI
**A (F) → PRI (C)**	.19	-.01	.40
**C (M) → PRI (C)**	.29	.11	.45
**Gf (M) → VCI (C)**	-.04	-.26	.23
**Gf (M) → PRI (C)**	-.01	-.22	.21
**Gs (M) → PSI (C)**	.28	.10	.44
**Gsm (M) → WMI (C)**	.24	-.06	.51
**Gsm (F) → WMI (C)**	.21	.02	.42
**Gv (F) → PRI (C)**	.15	-.03	.32
**Sex (C) → VCI (C)**	.33	-.14	.87
**Sex (C) → WMI (C)**	-.15	-.55	.23
**Edu (M) →VCI (C)**	.18	-.06	.38
**Edu (M) → PRI (C)**	.20	.00	.42
**Edu (M) → PSI (C)**	.07	-.12	.26
**Edu (F) → PSI (C)**	-.17	-.38	.03
**Sex (C) × Gsm (M) → WMI (C)**	-.16	-.53	.27
**Sex (C) × Edu (M) → VCI (C)**	.43	-.03	.80

Legend: O = Openness; VCI = Verbal Comprehension Index; PRI = Perceptual Reasoning Index; WMI = Working Memory Index; PSI = Processing Speed Index; Gs = processing speed; Gc = Comprehension-Knowledge; Gf = Fluid reasoning; Gv = Visual processing; Gsm = Short-Term memory; A = Agreeableness; C = Conscientiousness; (C) = Children variables; (M) = Mother variables; (F) = Father variables; B = regression coefficients; 5% LLCI = Lower Limit Confidence Interval; 95% ULCI = Upper Limit Confidence Interval.
